# Supplementary material for: Naive Pluripotent Stem Cells Exhibit Phenotypic Variability that Is Driven by Genetic Variation
Source: Cell Stem Cell. 2020 Sep 3;27(3):470–481.e6. doi: 10.1016/j.stem.2020.07.019 (PMC7487768; doi:10.1016/j.stem.2020.07.019)
Supplement: Document S1. Figures S1–S4 and Table S1 [file mmc1.pdf]

Cell Stem Cell, Volume *27*

Supplemental Information

Naive Pluripotent Stem Cells Exhibit Phenotypic Variability that Is Driven by Genetic Variation

Daniel Ortmann, Stephanie Brown, Anne Czechanski, Selcan Aydin, Daniele Muraro, Yuanhua Huang, Rute A. Tomaz, Anna Osnato, Giovanni Canu, Brandon T. Wesley, Daniel A. Skelly, Oliver Stegle, Ted Choi, Gary A. Churchill, Christopher L. Baker, Peter J. Rugg-Gunn, Steven C. Munger, Laura G. Reinholdt, and Ludovic Vallier

Supplementary information:

Figure S1.

Genetic background defines the transcriptional profile of naïve mESC. Related to Figure 1.

A

Strain	Cell Line	Karyotype	Germline Transmission
PWD/PhJ	401	70% XY	63%
PWD/PhJ	403	70% XY	22%
PWD/PhJ	398	70% XY	n/a
CAST/EiJ	77	74% XY	100%
CAST/EiJ	78	81% XY	100%
CAST/EiJ	80	80% XY	100%
C57BL/6J	49	95% XY	100%
C57BL/6J	139	100% XY	n/a
C57BL/6J	146	95% XY	n/a
C3H/HeJ	386	80% XY	90%
C3H/HeJ	388	77% XY	81%
C3H/HeJ	389	67% XY	n/a

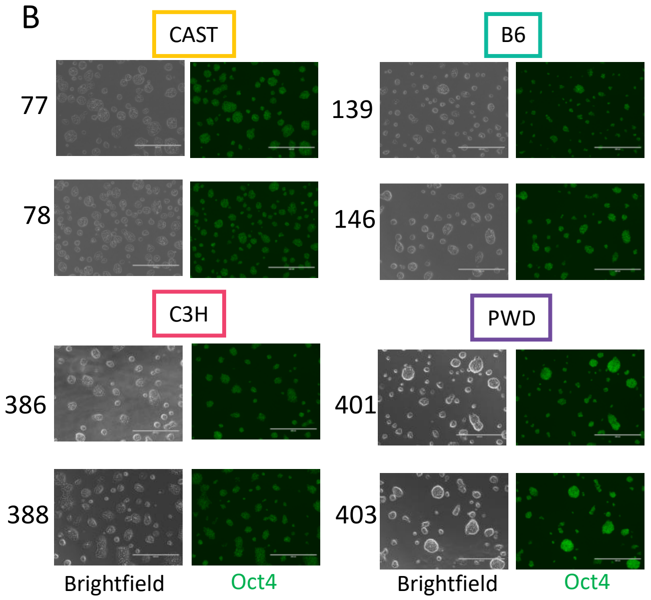

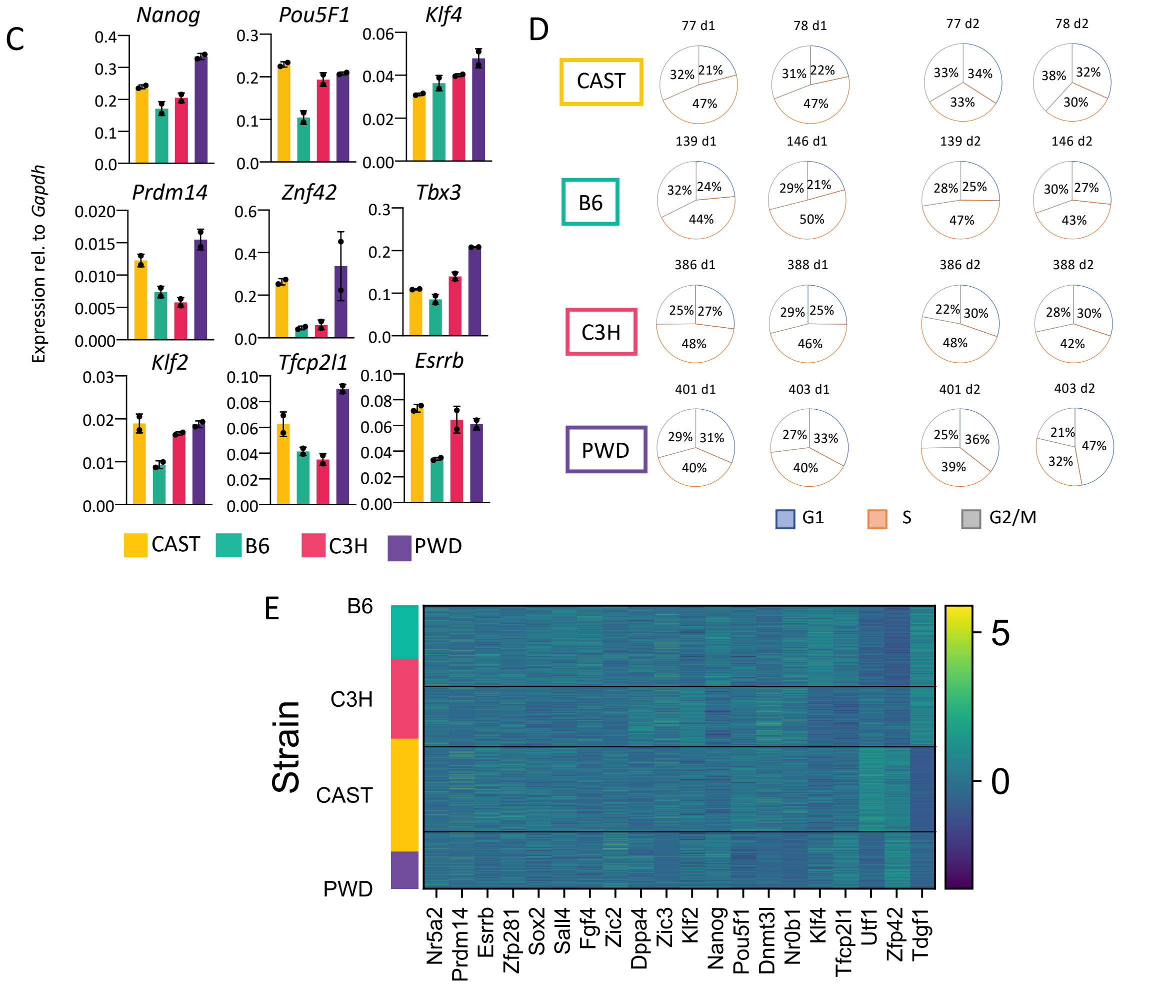

Supplementary figure 1 legend

A. Karyotype and chimera formation information for the lines used in this study.
B. Representative bright field and immunocytochemistry images of cells growing in 2iL. Number indicates the specific line shown.
C. Gene expression analysis for selected ground state/pluripotent markers. Data shown are the mean of 2 replicates per genetic background, 1 line per background. Error bars represent standard deviation of the means.
D. Cell cycle profiles over 2 days in 2iL condition. Data shown are representative of two independent experiments. Number indicates the specific line shown.
E. Heat map of selected z-scored normalised markers generated from Single Cell transcriptomic data.

Figure S2.

Genetic background influences early differentiation capacity of naïve mESCs. Related to Figure 2.

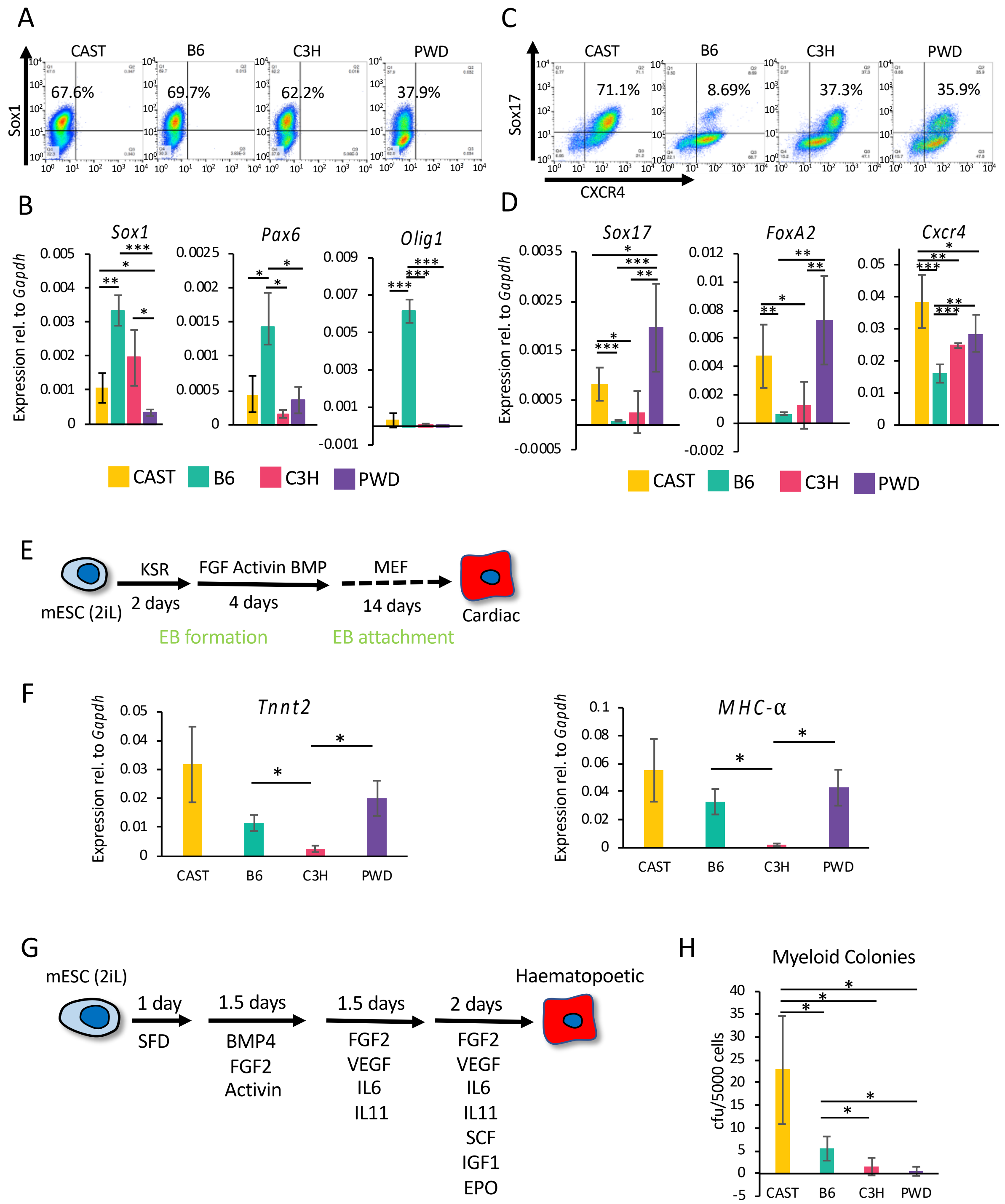

Supplementary Figure 2 Legend

A. Representative flow cytometry analysis of SOX1 after 6 days of undirected differentiation.
B. Gene expression analysis of key neural markers after 2 days of undirected differentiation. Data shown are from 2 differentiations for each genetic background, 2 lines per background. Error bars represent standard deviation of the means. Two tailed T-test *$p<0.05$, **$p<0.005$, ***$p<0.0005$.
C. Representative flow cytometry analysis of SOX17 and CXCR4 after 6 days of differentiation towards endoderm.
D. Gene expression analysis of key endodermal markers after 6 days of directed differentiation towards endoderm. Data shown are from 2 independent differentiations for each genetic background, 2 lines per genetic background. Error bars represent standard deviation of the means. Two tailed T-test *$p<0.05$, **$p<0.005$, ***$p<0.0005$.
E. Schematic representation of the cardiac differentiation process.
F. Gene expression analysis of key cardiac markers at the end of the differentiation process. Data shown are from 2 independent differentiations for each genetic background, 2 lines per genetic background. Error bars represent standard errors of the means. Two tailed T-test *$p<0.05$.
G. Schematic representation of the haematopoetic differentiation process.
H. Colony forming assay assessing haematopoetic differentiation. Data shown are from 1 differentiation for each genetic background, 2 lines per genetic background. Error bars represent standard deviation of the means. Two tailed T-test *$p<0.05$.

Figure S3.

Transition from mESCs to EpiLCs varies between genetic backgrounds. Related to Figure 3.

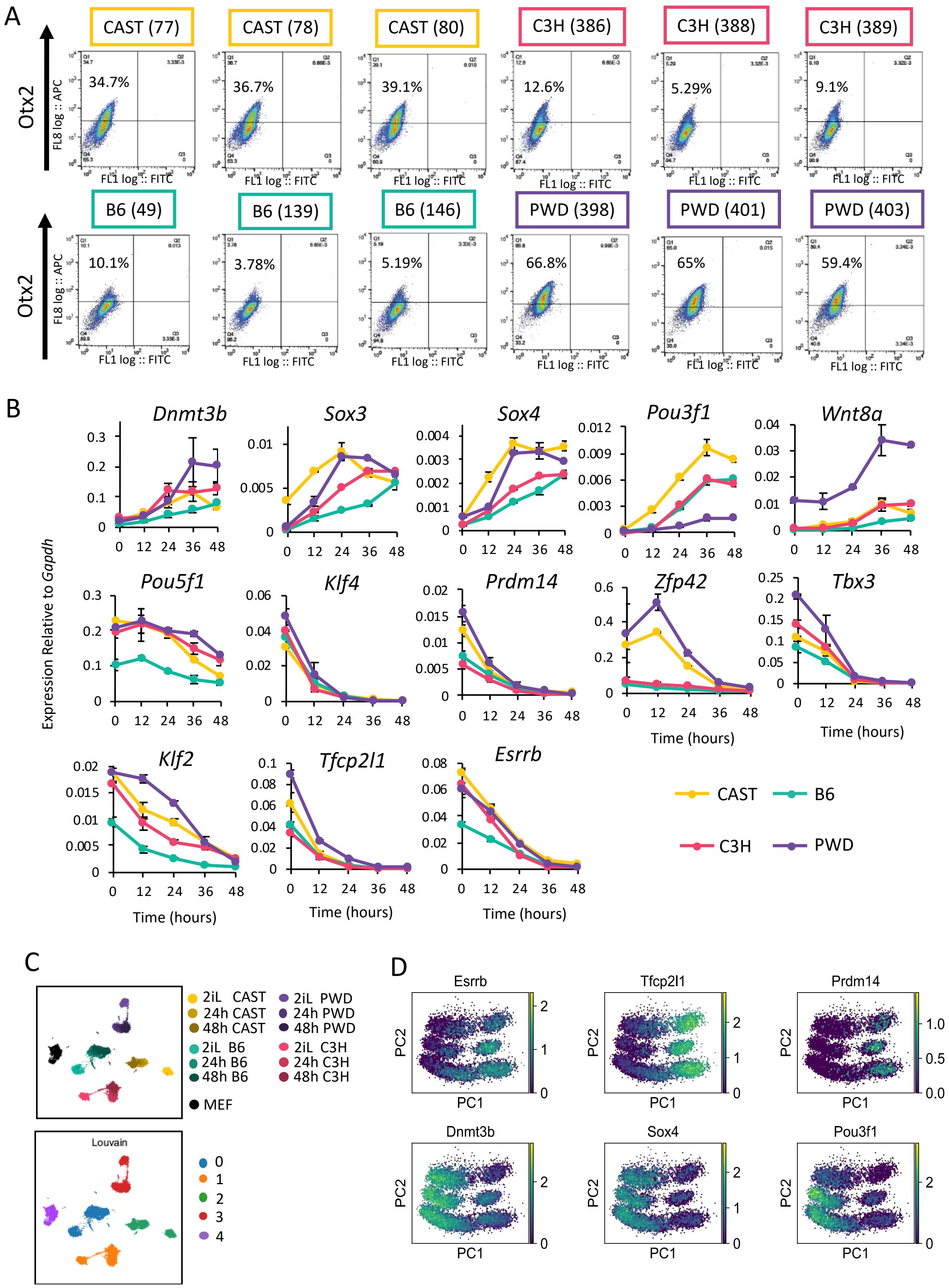

Supplementary figure 3 legend

A. Flow cytometry plots for OTX2 after 2 days of undirected differentiation in N2B27. Plots shown are from 1 differentiation, number in brackets indicates the specific line shown.
B. Gene expression analysis of selected markers during 2 days of undirected differentiation in N2B27. Error bars represent standard deviation of the means, 1 line per genetic background.
C. UMAP and Louvain clustering using 300 PCs (51.2% variance explained) during 2 days of undirected differentiation in N2B27. 5 clusters partition strains and negative control (MEF).
D. Log-normalised gene expression values of selected differentiation markers.

Figure S4.

Signalling activity is influenced by genetic background. Related to Figure 4.

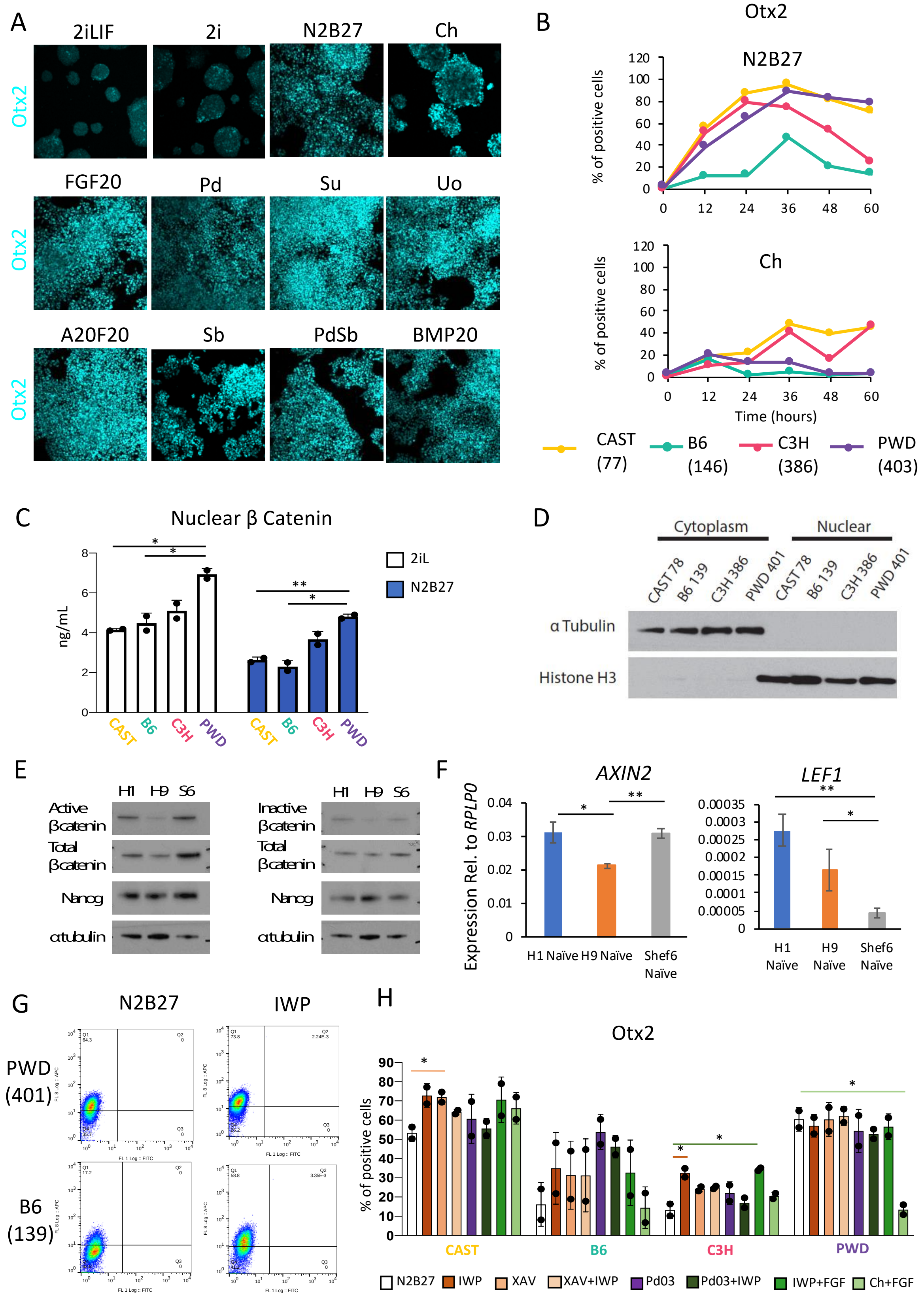

Supplementary Figure 4 Legend

A. Representative immunocytochemistry images for Otx2 after 2 days in the indicated culture conditions. Images shown are from the CAST background.
B. Quantification of flow cytometry analysis for Otx2 over a 60 hour time course in the indicated culture conditions. Number in brackets indicates the line shown.
C. ELISA for total β Catenin in a nuclear enriched cellular fraction after 1 day in the indicated culture conditions. Data shown are the mean from 2 lines per genetic background. Error bars represent standard deviation of the means. Two tailed T-test *$p<0.05$, **$p<0.005$.
D. Western blot for cytoplasmic and nuclear markers after cell fractionation. Number in brackets indicates the line shown.
E. Western blot for active and total β Catenin in human naive state cells derived from 3 different genetic backgrounds.
F. Gene expression analysis of selected WNT signalling target genes in human naïve state cells. Data shown are the mean from 3 replicates for each genetic background. Error bars represent standard deviation of the means. Two tailed T-test *$p<0.05$, **$p<0.005$.
G. Representative flow cytometry plots for Otx2 after 2 days in the indicated culture conditions. Number in brackets indicates the line shown.
H. Quantification of flow cytometry analysis for Otx2 after 2 days in the indicated culture conditions. Data shown are the mean from 2 lines per genetic background. Error bars represent standard deviation of the means. Two tailed T-test *$p<0.05$.

Table S1.

a. Quantitative PCR primer sequences. Related to STAR Methods section.
b. PAGA analysis of single cell RNA-seq time course. Related to Figure S3c.

Supplementary table 1a:

Gene Symbol	Forward Primer (5'-3')	Reverse Primer (5'-3')
Gapdh	ACACATTGGGGGTAGGAACA	AACTTTGGCATTGTGGAAGG
Zfp42	CAAGGAGGAAATAGGTAGAGCGCA	ACTTGGAGGCAGCACAGTGA
Nanog	CACAGTTTGCCTAGTTCTGAGG	GCAAGAATAGTTCTCGGGATGAA
Pou5F1	ATCACTCAATCCGCCAATCAGC	GCCGGTTACAGAACCATACTCG
Sox2	CACATGGCCCAGCACTACC	CACATGTGCGACAGGGGC
Klf4	AAGAACAGCCACCCACACTT	GGTAAGGTTTCTCGCCTGTG
Prdm14	GCATATACCCTACCCGCTTTC	CAAACGGATTGGAGGTTGAT
Otx2	TATCTAAAGCAACCGCCTTACG	AAGTCCATACCCGAAGTGGTC
Dnmt3b	GCCCATGCAATGATCTCTCT	CCAGAAGAATGGACGGTTGT
Pou3F1	GATCCAGAATGCGCCAACTCAC	CCTCTCTTCGTCCATTCTCCCG
FGF5	GGGATTGTAGGAATACGAGGAGTT	TGGCACTTGCATGGAGTTT
Sox1	TGTAATCCGGGTGTTCCTTC	AGTGGAAGGTCATGTCCGAG
Axin2	GAGAGTGAGCGGCAGAGC	CGGCTGACTCGTTCTCCT
Lef1	TCCTGAAATCCCCACCTTC	ACCCGTGATGGGATAAACAG
Wnt5a	CTGGAGGTGCCATGTCTTCC	TCGGCTGCCTATTTGCATCA
Pdgrfa	AAGACCTGGGCAAGAGGAAC	GAACCTGTCTCGATGGCACT
Gata6	GGTCTCTACAGCAAGATGAATGG	TGGCACAGGACAGTCCAAG
Sox7	GCGGAGCTCAGCAAGATG	GGGTCTCTTCTGGGACAGTG
Lama1	AGGTCTGCGTTGAGTGTTCTG	CAGTACTATGCCGTCAGCGAT
Sparc1	AGGGCCTGGATCTTCTTTCTC	CAAATTCTCCCATTTCCACCT
Tbx3	CTGCCCTTCCACCTCCAACA	GGGGCGCATGCTGTTCAAAT
Klf2	AAGAGCTCGCACCTAAAGGC	CGCATCCTTCCCAGTTGC
Tfcp2l1	GGGGACTACTCGGAGCATCT	TTCCGATCAGCTCCCTTG
Esrrb	GATGCTGAAGGAAGGTGTGC	GGCTTTTTAGCAGGTGGGGA
Tnnt2	CAACATGATGCACTTTGGAGGGT	TCGCAGAACGTTGATTTCGTATT
MHC alpha	GCCCAGTACCTCCGAAAGTC	GCCTTAACATACTCCTCCTTGTC
FoxA2	CCCTACGCCAACATGAACTCG	GTTCTGCCGGTAGAAAGGGA
Cxcr4	CTTCTGGGCAGTTGATGCCAT	CTGTTGGTGGCGTGGACAAT
Sox3	CTGGAAACTGCTGACCGATG	TCCGGGTACTCCTTCATGTG
Sox4	CTCAAGGACAGCGACAAGATTCC	AGCCATGTGCTTGAGGCG
Wnt8a	CATGTACGCAGTCACCAAGAA	CATCCTTCCCTTTCTCCAAAC
hs *AXIN2*	GCTCTGTTTGTCTTAAAGGTCT	AGGAACTGTCATTTCCACGAAAG
hs *LEF1*	TGAGCCTCGAGAAGAAAAACCG	CCACGCTGGAGATGTCCGTT
hs *RPLP0*	CAACTGTTGCATCAGTACCCCATTCT	ACTCTTCCTTGGCTTCAACCTTAGCT
Sox17	QuantiTect Mm_Sox17_1_SG	QT00160720

Supplementary Table 1b:

	2iLCAST	24hCAST	48hCAST	2iLB6	24hB6	48hB6	2iLPWD	24hPWD	48hPWD	2iLC3H	24hC3H	48hC3H	MEF
2iLCAST	0	4.43	1.09	0.37	0	0	2.92	0.19	0	0	0	0.18	0.56
24hCAST	4.43	0	100	0.14	0.3	0.67	0.48	1.88	0.28	0	0	0.39	0
48hCAST	1.09	100	0	0	0	0.72	0.7	1.558	0.22	0	0	2.28	0
2iLB6	0.37	0.14	0	0	13.66	6.94	0.32	0	0.23	0.67	0	0.32	0
24hB6	0	0.3	0	13.66	0	100	0	0.06	0.26	0.13	0.82	0	0
48hB6	0	0.67	0.72	6.94	100	0	1.05	3.04	0.08	0	1.46	8.11	0
2iLPWD	2.92	0.48	0.7	0.32	0	1.05	0	14.97	9.33	0	0	0.46	0
24hPWD	0/19	1.88	1.58	0	0.06	3.04	14.97	0	100	0.09	0.27	2.65	0
48hPWD	0	0.28	0.22	0.23	0.26	0.08	9.33	100	0	0	0	0.26	0
2iLC3H	0	0	0	0.67	0.13	0	0	0.09	0	0	6.48	2.67	0
24hC3H	0	0	0	0	0.82	1.46	0	0.27	0	6.48	0	100	0
48hC3H	0.18	0.39	2.28	0.32	0	8.11	0.46	2.65	0.26	2.67	100	0	0
MEF	0.56	0	0	0	0	0	0	0	0	0	0	0	0
