# Supplementary material for: Naive Pluripotent Stem Cells Exhibit Phenotypic Variability that Is Driven by Genetic Variation
Source: Cell Stem Cell. 2020 Sep 3;27(3):470–481.e6. doi: 10.1016/j.stem.2020.07.019 (PMC7487768; doi:10.1016/j.stem.2020.07.019)
Supplement: Document S2. Article plus Supplemental Information [file mmc5.pdf]

Short Article

Cell Stem Cell

# Naive Pluripotent Stem Cells Exhibit Phenotypic Variability that Is Driven by Genetic Variation

## Graphical Abstract

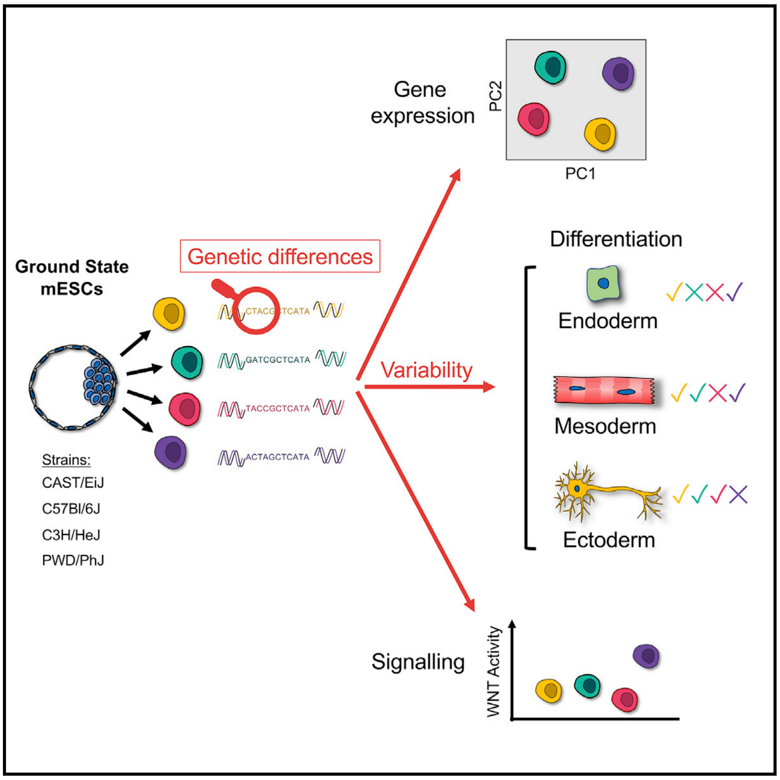


## Authors

Daniel Ortmann, Stephanie Brown, Anne Czechanski, ..., Steven C. Munger, Laura G. Reinholdt, Ludovic Vallier

## Correspondence

daniel.ortmann@gmail.com (D.O.), lv225@cam.ac.uk (L.V.)

## In Brief

Ortmann et al. show that ground-state pluripotent stem cells exhibit variability in gene expression and differentiation propensity that is associated with their genetic background. This variability is linked to differences in major signaling pathway activity, showing that analyses of expression quantitative trait loci enable identification of causal genetic elements.

## Highlights

- Ground-state mESCs are variable in gene expression and capacity of differentiation
- Genetic background contributes to ESC variability even after epigenetic resetting
- Signaling pathway activity is influenced by genetic traits
- Analysis of eQTL identifies genetic elements causing variability

Ortmann et al., 2020, Cell Stem Cell *27*, 470–481
September 3, 2020 © 2020 The Authors. Published by Elsevier Inc.
https://doi.org/10.1016/j.stem.2020.07.019

CellPress

CellPress
OPEN ACCESS

Cell Stem Cell

Short Article

# Naive Pluripotent Stem Cells Exhibit Phenotypic Variability that Is Driven by Genetic Variation

Daniel Ortmann,[1,2,10,*] Stephanie Brown,[1,2,10] Anne Czechanski,[3] Selcan Aydin,[3] Daniele Muraro,[1,2,8] Yuanhua Huang,[4,5] Rute A. Tomaz,[1,2] Anna Osnato,[1,2] Giovanni Canu,[1,2] Brandon T. Wesley,[1,2] Daniel A. Skelly,[3] Oliver Stegle,[5,6,7] Ted Choi,[3] Gary A. Churchill,[3] Christopher L. Baker,[3] Peter J. Rugg-Gunn,[1,9] Steven C. Munger,[3] Laura G. Reinholdt,[3] and Ludovic Vallier[1,2,11,*]

[1]Wellcome Trust and MRC Cambridge Stem Cell Institute, University of Cambridge, Cambridge, UK
[2]Department of Surgery, University of Cambridge, Cambridge, UK
[3]Jackson Laboratory, Bar Harbor, ME, USA
[4]Department of Clinical Neurosciences, University of Cambridge, Cambridge, UK
[5]European Molecular Biology Laboratory, European Bioinformatics Institute, Wellcome Genome Campus, Hinxton, UK
[6]European Molecular Biology Laboratory, Genome Biology Unit, Heidelberg, Germany
[7]Division of Computational Genomics and Systems Genetics, German Cancer Research, Center (DKFZ), Heidelberg, Germany
[8]Wellcome Sanger Institute, Wellcome Genome Campus, Hinxton, UK
[9]Epigenetics Programme, Babraham Institute, Cambridge, UK
[10]These authors contributed equally
[11]Lead Contact
*Correspondence: daniel.ortmann@gmail.com (D.O.), lv225@cam.ac.uk (L.V.)
https://doi.org/10.1016/j.stem.2020.07.019

## SUMMARY

Variability among pluripotent stem cell (PSC) lines is a prevailing issue that hampers not only experimental reproducibility but also large-scale applications and personalized cell-based therapy. This variability could result from epigenetic and genetic factors that influence stem cell behavior. Naive culture conditions minimize epigenetic fluctuation, potentially overcoming differences in PSC line differentiation potential. Here we derived PSCs from distinct mouse strains under naive conditions and show that lines from distinct genetic backgrounds have divergent differentiation capacity, confirming a major role for genetics in PSC phenotypic variability. This is explained in part through inconsistent activity of extra-cellular signaling, including the Wnt pathway, which is modulated by specific genetic variants. Overall, this study shows that genetic background plays a dominant role in driving phenotypic variability of PSCs.

## INTRODUCTION

Phenotypic variability among human pluripotent stem cell (hPSC) lines affects their capacity of differentiation, which impedes the development of universal protocols for the production of cell types with clinical interest. This phenomenon has been described and characterized extensively for both human embryonic stem cells (hESCs) and induced pluripotent stem cells (iPSCs) (Bock et al., 2011; Boulting et al., 2011; Osafune et al., 2008). Several studies have shown that variations in transcriptome profiles are more prominent among lines derived from different donors (DeBoever et al., 2017; Rouhani et al., 2014), and large-scale studies have identified candidate expression quantitative trait loci (eQTL) that could induce phenotypic divergence of hPSC lines (Alasoo et al., 2018; Carcamo-Orive et al., 2017; Cuomo et al., 2020; Kilpinen et al., 2017). However, functional validations have not yet uncovered the interplays among these genetic variants and the mechanisms controlling differentiation. More important, complementary reports have shown that epigenetic regulations such as DNA methylation (Butcher et al., 2016; Nazor et al., 2012; Nishizawa et al., 2016) could be an alternative explanation for this divergence. Distinguishing between these two sources of variation is difficult with hPSCs because of their genetic diversity and the complexity of their epigenetic state. Mouse epiblast stem cells (mEpiSCs) (the equivalent of hPSCs; Brons et al., 2007) have also been shown to display both variability among lines and heterogeneity in gene expression (Bernemann et al., 2011; Han et al., 2010), suggesting that similar mechanisms could exist across species. On the other hand, mouse embryonic stem cells (mESCs) can be grown in chemically defined ground-state culture conditions (Nichols and Smith, 2009; Ying et al., 2008) that minimize the influence of epigenetic variability by lowering histone and DNA methylation (Leitch et al., 2013; Marks et al., 2012). This resetting could decrease the impact of epigenetic factors and thus overcome variability in differentiation potential among different mESC lines. However, systematic studies are still lacking to fully demonstrate this possibility. Of note, a number of studies have developed ground-state conditions for the production of naive hPSCs (Gafni et al., 2013; Takashima et al., 2014; Theunissen et al., 2014).

 © 2020 The Authors. Published by Elsevier Inc.
This is an open access article under the CC BY license (http://creativecommons.org/licenses/by/4.0/).

CellPress
OPEN ACCESS

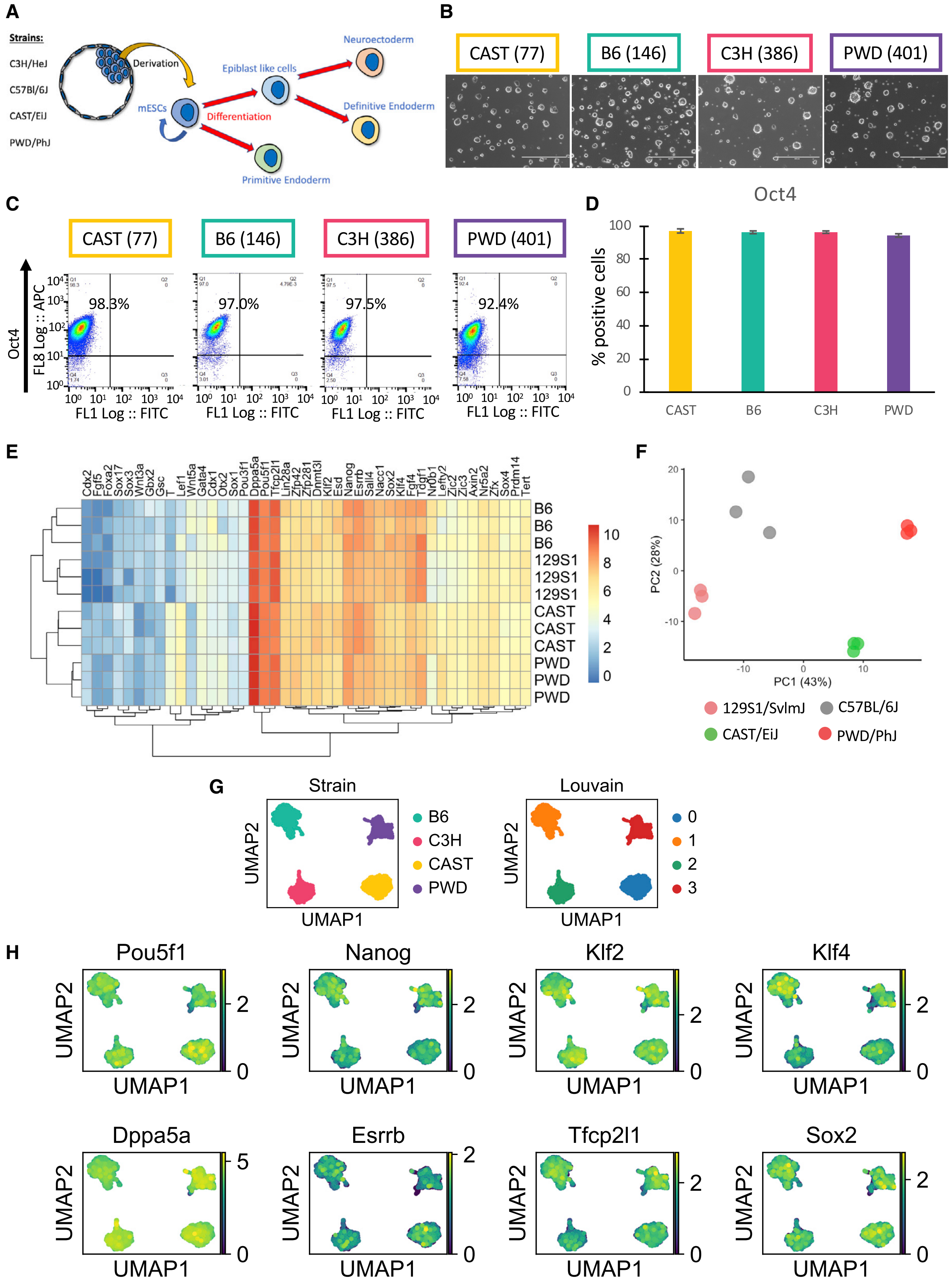


**Figure 1. Genetic Background Defines the Transcriptional Profile of Naive mESCs**

(A) Graphical representation of mESC ground state lines showing differentiation toward different defined lineages.

(B) Representative bright-field images of cells growing in 2iL. Number in brackets indicates the specific line shown.

(legend continued on next page)

However, the resulting lines are often challenging to grow, are genetically variable in some conditions, and require an initial capacitation step prior to multi-lineage differentiation (Rostovskaya et al., 2019). Therefore, the hypothesis that ground-state culture conditions and the associated epigenetic resetting can overcome phenotypic variability observed in genetically diverse pluripotent stem cell lines remains to be experimentally tested.

Here, we decided to address this issue by taking advantage of 12 inbred mESC lines from four common laboratory strains, along with transcriptome data from 185 outbred mESC lines (Diversity Outbred) from the Collaborative Cross (Churchill et al., 2004). These lines were assessed for their potential to generate different cell types, and we observed that naive pluripotent stem cells derived from the same strain display a consistent capacity of differentiation. However, cell lines with different genetic background display strikingly divergent capacities to produce specific cell types. Furthermore, Wnt signaling regulation and activity varies among different strains, both in pluripotency and during differentiation. Finally, we identified eQTL suggesting that the activity of major signaling pathways could be controlled by genetic variants. In conclusion, our study demonstrates that genetic mechanisms strongly influence the efficacy of differentiation *in vitro*, and resetting the epigenetic state of pluripotent stem cells might not be sufficient to bypass phenotypic variability.

## RESULTS

### Genetic Background Defines the Transcriptional Profile of Naive mESCs

Individual mESC lines were derived from different blastocysts of four different strains (CAST/EiJ [CAST], C57BL/6J [B6], C3H/HeJ [C3H], and PWD/PhJ [PWD]; Figure 1A). These lines displayed a normal karyotype, formed chimeras, and showed germline contribution (Skelly et al., 2020 [in this issue of *Cell Stem Cell*]; Czechanski et al., 2014; Figure S1A). For this study, naive mESCs were grown in ground-state conditions in the presence of LIF, the GSK3 ($\alpha$ and $\beta$) inhibitor CHIR99021, and the MEK inhibitor PD0325901, as described (Ying et al., 2008). All lines grew as dome-shaped colonies characteristic of mouse pluripotent stem cells (Figure 1B). They also homogeneously expressed pluripotency markers and shared a similar cell cycle profile (Figures 1C, 1D, and S1B–S1D). Interestingly, bulk RNA sequencing (RNA-seq) confirmed that conventional pluripotency markers were expressed at similar levels among naive mESC lines of the same strain, with few exceptions (Figure 1E; Skelly et al., 2020). However, a broad number of genes were differentially expressed (Figure 1E) among lines from different genetic backgrounds. Similarly, unsupervised principal-component analysis (PCA) of global gene expression profiles showed that cells from different strains have divergent transcriptomic profiles (Figure 1F). A list of genes contributing to this divergence can be found in Table S2. Interestingly, Gene Ontology analysis of driver genes for PC1 related to molecular function yielded the following significant contributions: calcium ion binding, transmembrane signaling receptor activity, signaling receptor activity, molecular transducer activity, and endopeptidase activity. This hints at differences in signaling activity of several pathways in different genetic backgrounds. Importantly, single-cell transcriptional profiling revealed that this effect is not caused by the presence of sub-populations of cells in our culture conditions. On the contrary, mESCs grown in ground-state conditions represent a homogeneous population expressing canonical pluripotency markers while displaying expression profiles specific for each genetic background (Figures 1G, 1H, and S1E). These results, combined with the analysis presented by Skelly et al. (2020), show that the genetic background of naive mESCs is a primary determinant of their transcriptional profiles, paralleling previous observations obtained with hPSCs (Rouhani et al., 2014).

### Genetic Background Influences Early Differentiation Capacity of Naive mESCs

In order to further investigate the functional impact of genetic background, three independently derived naive mESC lines from each mouse strain were differentiated simultaneously using the same culture conditions to minimize experimental variations. We first tested their differentiation capacity in an unguided manner by simply withdrawing 2i and LIF (Figure 2A). In these conditions, mESCs differentiate toward the neurectoderm lineage without the need for additional factors (Tropepe et al., 2001). Efficiency of differentiation was measured by quantifying the fraction of cells expressing the neuroectoderm marker SOX1 after 6 days. All the lines tested were able to produce neuroectoderm cells, confirming their pluripotent state. However, the fraction of SOX1-expressing cells varied substantially among lines from different genetic backgrounds (Figures 2B, 2C, S2A, and S2B). B6 mESCs showed the highest neuroectoderm capacity, whereas the PWD lines were the least efficient in producing SOX1-expressing cells. Overall, genetic background had a strong and statistically significant effect on differentiation to neuroectoderm (paired t test; Figures 2C and S2B). Thus, naive mESCs display different capacities of differentiation toward the "default" neuroectoderm lineage, and this variability seems to be defined by their genetic background.

We then decided to probe the potential of naive ESC lines for a different cell fate controlled by inductive cues. For that, cells were grown for 6 days in culture conditions promoting definitive endoderm (Figure 2D), and differentiation efficiency was quantified by measuring the fraction of cells expressing SOX17. Interestingly, CAST mESCs produced the largest number of SOX17$^{+}$ cells, while B6 mESCs showed the lowest differentiation efficiency (Figures 2E, 2F, S2C, and S2D). Again, the genetic

(C) Representative flow cytometry plots for Oct4 in cells growing in 2iL. Number in brackets indicates the specific line shown.
(D) Quantitation of flow cytometry for Oct4 in cells growing in 2iL. Data shown represent a minimum of five samples from each genetic background, two lines per background. Error bars represent the standard error of the means.
(E) Heatmap of selected markers generated from RNA-seq data. Data shown are from three independently derived lines for each genetic background.
(F) Principal-component analysis plot of transcriptome data. Data shown are from three independently derived lines for each genetic background.
(G) UMAP plot generated from single-cell data. Strains are partitioned into four clusters (Louvain method).
(H) UMAP plot showing log-normalized gene expression of selected markers.

CellPress
OPEN ACCESS

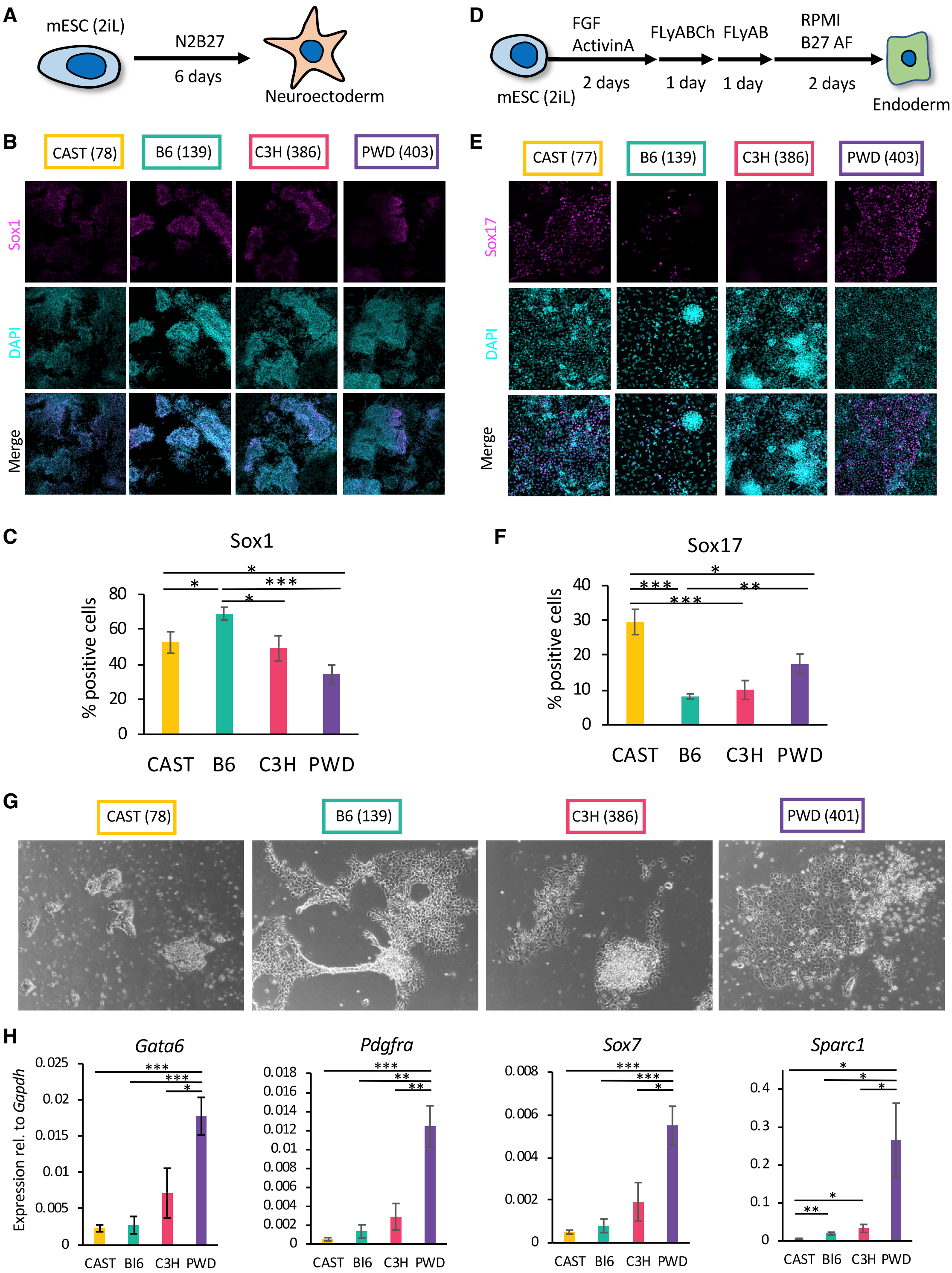


**Figure 2. Genetic Background Influences Early Differentiation Capacity of Naive mESCs**

(A) Schematic representation of undirected differentiation of mESC lines.

(B) Representative immunocytochemistry images for SOX1 after 6 days of undirected differentiation. Number in brackets indicates specific line shown.

(C) Quantification of flow cytometry data for SOX1 after 6 days of undirected differentiation. Data shown are from six independent differentiations for each genetic background, two lines per background. Error bars represent standard errors of the means. *p < 0.05 and ***p < 0.0005 (two-tailed t test).

(D) Schematic representation of differentiation toward definitive endoderm.

(E) Representative immunocytochemistry images for SOX17 after 6 days of endoderm differentiation. Number in brackets indicates specific line shown.

(legend continued on next page)

background had a significant effect on the differentiation efficiency (paired t test; Figure 2F).

Importantly, mESCs do not generate endoderm cells directly, as only late epiblast cells have this capacity. Consequently, germ-layer specification can be achieved only after transition through different pluripotent states (Smith, 2017). On the other hand, mESCs can directly give rise to primitive endoderm. Thus, we decided to test the capacity of naive ESC lines for this differentiation by taking advantage of a protocol based on defined culture conditions (Anderson et al., 2017). Interestingly, CAST mESCs displayed a limited capacity to form primitive endoderm, while PWD mESCs were able to produce cells with distinctive morphology (Figure 2G) expressing *Gata6*, *Sox7*, *Pdgfrα*, and *Sparc* (Figure 2H). Of note, production of primitive endoderm cells was challenging, with most naive ESC lines suggesting that this direct route of differentiation could benefit from further optimization. Nonetheless, these analyses confirm that genetic background influence the capacity of naive mESCs to directly generate differentiated progenies.

Finally, we decided to investigate if this variability could also affect the ability of naive mESC lines to generate more mature cell types. For that, naive mESC lines were differentiated into cardiomyocytes and hematopoietic cells (Ditadi et al., 2015). Interestingly, CAST mESCs displayed the higher capacity to generate cardiac and blood cells, whereas the PWD strain seems to be giving rise to cardiomyocytes but not hematopoietic cells under the chosen conditions (Figures S2E–S2H). Thus, variability among naive ESC lines is not limited to early stages of differentiation. In sum, our data suggest that naive mESC lines from different strains show different propensities for lineage priming and commitment under identical culture conditions. Specifically, B6 and C3H mESC lines showed the highest efficiency in generating ectoderm, CAST mESCs the highest capacity for definitive endoderm and mesoderm derivatives, and PWD mESCs the highest capacity to form primitive endoderm. Thus, the differentiation capacity of naive mESCs appears to be heavily influenced by their genetic background.

## Transition from mESCs to EpiLCs Varies among Genetic Backgrounds

We then opted to resolve the precise stage at which the differentiation capacity of mESC lines starts to diverge by studying the transition between the naive and the post-implantation epiblast state (EpiLCs; Buecker et al., 2014). mESCs were grown for 2 days in N2B27 medium in the absence of 2i LIF, and the proportion of cells expressing OTX2 was evaluated. This transcription factor is necessary to exit the naive state and is also required to protect EpiLCs from premature neuroectoderm specification (Acampora et al., 2013; Yang et al., 2014). A high proportion of OTX2-positive cells were detected in differentiating CAST and PWD mESCs, whereas B6 and C3H mESCs showed fewer positive cells (Figures 3A, 3B, and S3A). Interestingly, *Nanog* was quickly and homogeneously downregulated in all of the lines, while upregulation of canonical Epiblast markers such as *Fgf5* showed more variation (Figure 3C). Thus, the transition toward the post-implantation epiblast state seems to be affected rather than the exit from the naive state. A more detailed examination revealed that the dynamics of OTX2 expression differed among cell lines derived from different genetic backgrounds. Indeed, the number of cells positive for OTX2 (Figure 3D) peaked earlier and at lower levels for C3H mESCs, while CAST and PWD lines achieved the highest expression levels 12 h later. Finally, B6 lines were very inefficient in inducing OTX2 expression, as fewer than 50% of the cells were positive after 36 h. Importantly, this pattern of expression fits with the role of OTX2 in cell fate decision, as B6 mESC lines display the highest efficiency for neuroectoderm differentiation. These observations were reinforced by qPCR analyses of key marker genes showing that lines from different genetic backgrounds displayed substantial variability during the transition out of naive pluripotency (Figure S3B). We further confirmed these observations by performing single-cell RNA-seq during a short time course of differentiation in the N2B27 conditions. Clustering and PAGA analyses by accounting for 300 PCs showed transcriptional similarity within strains and relative strain similarity compared with a negative control (mouse embryonic fibroblasts; see Figure S3C; Table S1B; and https://github.com/theislab/paga), thereby confirming the pluripotent identity of the cell lines used in this study. PCAs (Figure 3E) showed that the biggest variance (PC1) is explained by differentiation (0–48 h), as cells exiting pluripotency (Figure S3D) drastically change their gene expression profile. The second major component (PC2) separates the different genetic backgrounds. Although the transcriptomic profile of B6 and C3H cells cluster together, those of CAST and PWD are clearly separate, reaffirming the substantial differences in gene expression driven by genetic backgrounds. The higher levels of *Otx2* induction in CAST and PWD cell line was confirmed in this dataset (Figure 3F). In addition, B6 and C3H lines display rapid upregulation of *Sox1* transcripts (Figure 3F), reflecting a higher propensity for neurectodermal differentiation. Taken together, these data suggest that variability in naive mESCs originates from their capacity to transition into post-implantation epiblast cells and from the expression of key regulators of this process such as OTX2.

## Signaling Activity Is Influenced by Genetic Background

To uncover the potential drivers of this variability, we tested the importance of different signaling pathways for the transition between naive mESCs and EpiLCs (Figures 4A and S4A). Neither ERK or TGF-β pathway modulation seemed to affect OTX2

(F) Quantification of flow cytometry data for SOX17 after 6 days of directed differentiation toward endoderm. Data shown are from seven independent differentiations for each genetic background, two lines per background. Error bars represent standard errors of the means. *$p < 0.05$, **$p < 0.005$, and ***$p < 0.0005$ (two-tailed t test).

(G) Representative bright-field images from each genetic background of cells differentiated for 12 days toward the primitive endoderm lineage. Number in brackets indicates specific line shown.

(H) Gene expression analysis of key markers after six days of directed differentiation toward primitive endoderm. Data shown are from five independent differentiations for each genetic background, two lines per genetic background. Error bars represent SD of the means. *$p < 0.05$, **$p < 0.005$, and ***$p < 0.0005$ (two-tailed t test).

CellPress
OPEN ACCESS

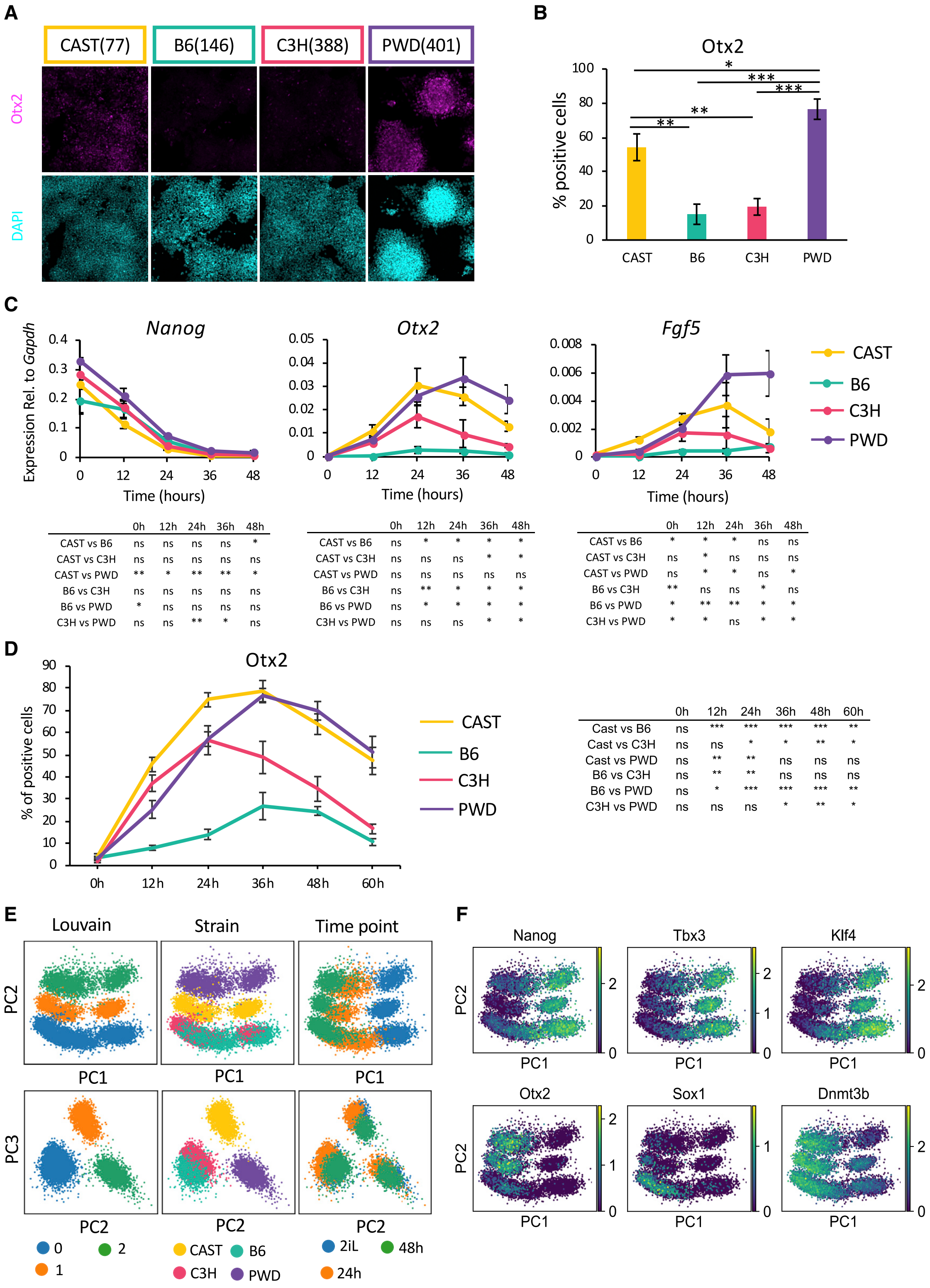


| | 0h | 12h | 24h | 36h | 48h |
|---|---|---|---|---|---|
| CAST vs B6 | ns | ns | ns | ns | * |
| CAST vs C3H | ns | ns | ns | ns | ns |
| CAST vs PWD | ** | * | ** | ** | * |
| B6 vs C3H | ns | ns | ns | ns | ns |
| B6 vs PWD | * | ns | ns | ns | ns |
| C3H vs PWD | ns | ns | ** | * | ns |

| | 0h | 12h | 24h | 36h | 48h |
|---|---|---|---|---|---|
| CAST vs B6 | ns | * | * | * | * |
| CAST vs C3H | ns | ns | ns | * | * |
| CAST vs PWD | ns | ns | ns | ns | ns |
| B6 vs C3H | ns | ** | * | * | * |
| B6 vs PWD | ns | * | * | * | * |
| C3H vs PWD | ns | ns | ns | * | * |

| | 0h | 12h | 24h | 36h | 48h |
|---|---|---|---|---|---|
| CAST vs B6 | * | * | * | ns | ns |
| CAST vs C3H | ns | * | ns | ns | ns |
| CAST vs PWD | ns | * | * | ns | * |
| B6 vs C3H | ** | ns | ns | * | ns |
| B6 vs PWD | * | ** | ** | * | * |
| C3H vs PWD | * | * | ns | * | * |

| | 0h | 12h | 24h | 36h | 48h | 60h |
|---|---|---|---|---|---|---|
| Cast vs B6 | ns | *** | *** | *** | *** | ** |
| Cast vs C3H | ns | ns | * | * | ** | * |
| Cast vs PWD | ns | ** | ** | ns | ns | ns |
| B6 vs C3H | ns | ** | ** | ns | ns | ns |
| B6 vs PWD | ns | * | *** | *** | *** | ** |
| C3H vs PWD | ns | ns | ns | * | ** | * |

**Figure 3. Transition from mESCs to EpiLCs Varies among Genetic Backgrounds**

(A) Representative immunocytochemistry images for Otx2 after 2 days of undirected differentiation in N2B27. Number in brackets indicates specific line shown.

(B) Quantification of flow cytometry data for OTX2 after 2 days of undirected differentiation in N2B27. Data shown are from two independent differentiations for each genetic background, three lines per background. Error bars represent standard errors of the means. *$p < 0.05$, **$p < 0.05$, and ***$p < 0.0005$ (two-tailed t test).

(legend continued on next page)

expression. On the other hand, addition of the GSK3 inhibitor CHIR99021 and subsequent increase of canonical Wnt β-catenin signaling led to a drastic reduction of OTX2-positive cells, suggesting a delay in the transition toward post-implantation pluripotent states (Figure S4B). We then asked whether a divergence in canonical Wnt signaling could explain the observed phenotypes during the transition to EpiLCs and assessed the levels of active β-catenin in the different naive mESC lines. These analyses revealed marked differences in the levels of active β-catenin among different genetic backgrounds (Figure 4B), with PWD mESCs showing the highest level of active β-catenin. Similar results were obtained upon the withdrawal of 2i LIF (in N2B27 basal medium), suggesting that the observed variation in Wnt signaling activity persisted after induction of differentiation (Figure 4B). Evaluation of the level of total β-catenin in the nuclear enriched fraction of the different lines also showed the same trend (Figures S4C and S4D). Importantly, these differences were confirmed using the TOP FLASH luciferase assay as a direct measure of canonical Wnt signaling activity (Figure 4C), as well as by qPCR for the levels of *Axin2*, a downstream target of canonical Wnt signaling and *Lef1* (Figure 4D). Thus, Wnt signaling activity appears to vary between mESC lines of different genetic backgrounds. Importantly, similar results were obtained with naive hPSC lines derived from three different individuals, indicating that variability in signaling pathways essential for differentiation could be shared between species (Figures S4E and S4F). To confirm the influence of this variation on differentiation, we investigated whether blocking Wnt signaling during differentiation of naive ESCs could be beneficial (ten Berge et al., 2011; Sugimoto et al., 2015). Indeed, addition of the PORCN inhibitor IWP2 increased the expression of OTX2 during the transition between naive to EpiLC state, especially in lines that showed lower levels of OTX2 in its absence (Figures 4E and S4G). Nevertheless, this increase did vary among mESC lines, suggesting that Wnt inhibition only partially decreases the variability in differentiation capacity. We also noted that the basal level of Wnt signaling activity did not correlate systematically with OTX2 expression. As an example, PWD mESC lines display high levels of Wnt activity while also expressing a high level of OTX2. Thus, transition from the naive state is likely to be controlled by complex interplays among several signaling pathways. This notion was reinforced further by a quantitative screen of inhibitors during the transition to EpiLCs (Figure S4H). Although IWP improved the generation of OTX2-positive cells in CAST, B6, and C3H backgrounds, inhibition of the FGF/MEKK pathway by Pd03 showed a strong effect only in the B6 background. Thus, these results show that composite extra-cellular signaling interactions could be influenced by genetic backgrounds in naive mESCs, thereby resulting in altered cell fate choices *in vitro*.

## eQTL Modulate Genes Associated with Wnt Pathway

Interestingly, we noticed that the expression of several Wnt pathway-related genes, including *Axin2* and *Lef1*, diverges among different naive human and mouse ESC lines (Figures 4D, S4G, and S4H). Of note, both Axin2 and Lef1 are effectors of the Wnt pathway and have an important function in regulating β-catenin activity (Figure 4C). Furthermore, single-cell transcriptional analysis in pluripotency conditions revealed differential expression of a broad number of Wnt signaling-related genes. The PWD background showed increased expression of some Wnt-related genes, such as AXIN2, LEF1, CSNK1E, GNAI1, NKD1, TCF7, and WNT5B (see Table S3 for a full list of differentially expressed genes). Lines from this genetic background also displayed the highest Wnt activity in the assays presented above. Thus, we hypothesized that the expression of Wnt regulators could be influenced by genetic variants specific to each mouse strain. To confirm this hypothesis, we took advantage of a large eQTL dataset mapped with RNA-seq and array genotyping from 185 Diversity Outbred mESC lines (Skelly et al., 2020) grown in culture conditions that partially destabilize the ground state (1i LIF, serum). We identified distant (trans) eQTL influencing the expression levels of hundreds of target genes, including a number of Wnt-related genes. Two of these suggestive eQTL "hotspots" or "trans-bands" were associated with variability across mESC lines in *Axin2* transcript levels, including a 7-Mb-wide eQTL trans-band on chromosome 3 (515 target genes with distant QTL LOD > 6) and a 2.3-Mb-wide eQTL trans-band on chromosome 5 (396 target genes with distant QTL LOD > 6; Figure 4F; Table S4). Ingenuity Pathway Analysis revealed that the groups of target genes modulated by these eQTL were significantly enriched for known members of the Wnt signaling pathway (Benjamini-Hochberg adjusted p values of 0.04 [Chr3] and 0.02 [Chr5]), reinforcing the role of genetic variation in potentially influencing Wnt activity. Moreover, estimates of the founder strain haplotype effects at these hotspots matched the split observed in Wnt pathway activity, where lines with PWD or CAST ancestry in the eQTL regions affected target gene transcript levels differently than lines with B6 ancestry (Figure 4G). Importantly, the presence of multiple eQTL for different genes associated with the Wnt pathway suggests that variability in signaling activity could originate from combinations of different variants, each having limited impact on their own that render functional validations challenging. Of note, the Wnt signaling pathway is not the only one associated with variable expression of its components. Indeed, genetic variation was also associated with the variable expression of *Fgfrl1* (or *Fgfr5*; Sleeman et al., 2001), a putative Fgf pathway antagonist. Our analysis found a strong local (*cis*) eQTL on chromosome 5 (LOD > 14; Figure 4H). These results suggest that complex interplays among a diversity of genetic variants dictate the level of

(C) Gene expression analysis of selected markers during 2 days of undirected differentiation in N2B27. Data shown are from two independent differentiations for each genetic background, two lines per background. Error bars represent SD of the means. Table directly below each marker shows two-tailed t test (*p < 0.05 and **p < 0.005).

(D) Quantification of flow cytometry data for OTX2 during 2 days of undirected differentiation in N2B27. Data shown are from two independent differentiations for each genetic background, two lines per background. Error bars represent standard errors of the means. Table to the right shows two-tailed t test (*p < 0.05, **p < 0.005, and ***p < 0.005).

(E) PCA and Louvain clustering during 2 days of undirected differentiation in N2B27. Percentage of variance explained: 3.9% for PC1 and 2.5% for PC2.

(F) Log-normalized gene expression values of pluripotency markers.

CellPress
OPEN ACCESS

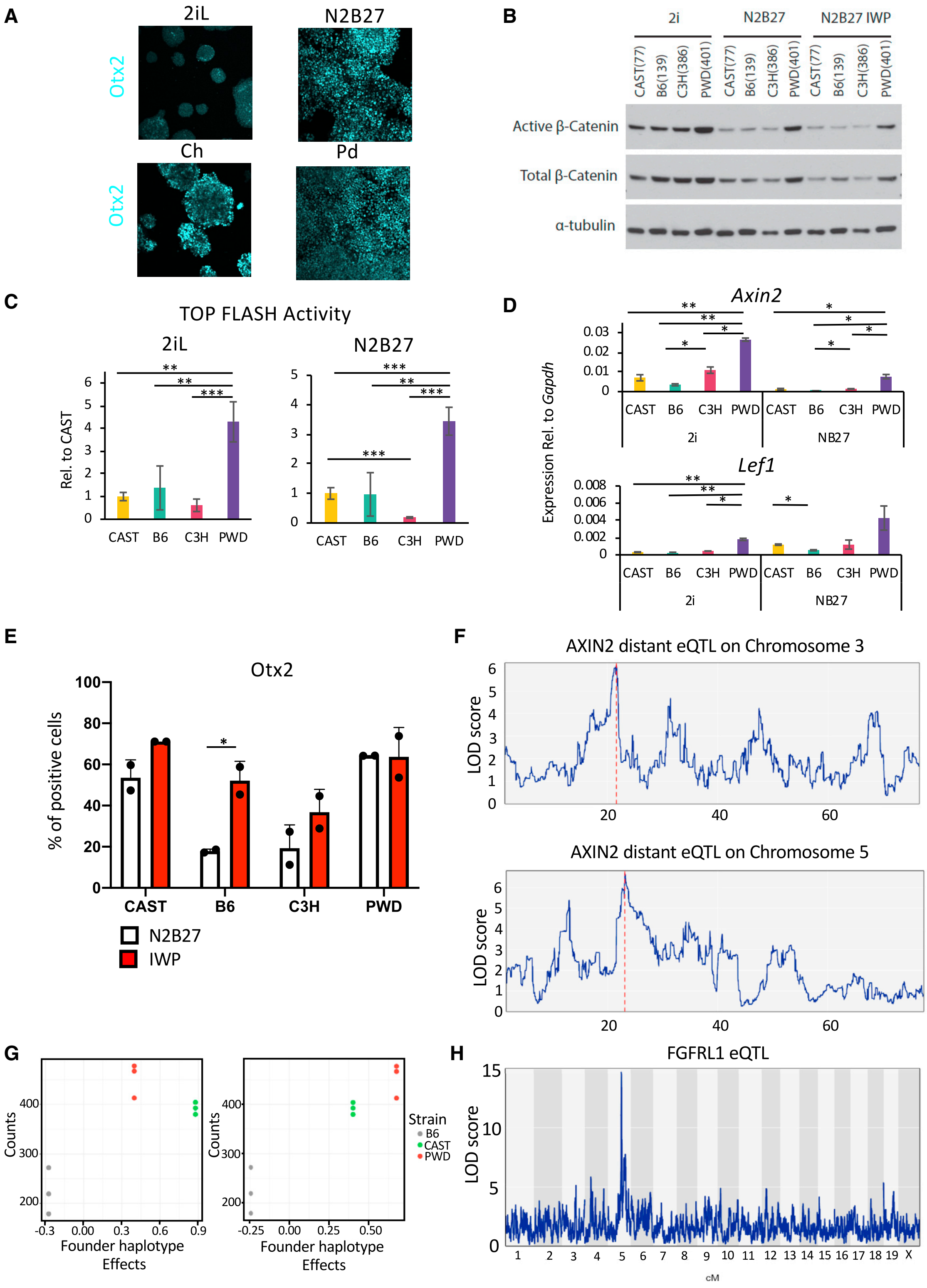


**Figure 4. Signaling Activity Is Influenced by Genetic Background**

(A) Representative immunocytochemistry images showing OTX2 levels after 2 days in the indicated culture conditions. Images shown are from the CAST background.

(B) Western blot for active and total β-catenin after 2 days in the indicated culture conditions. Number in brackets indicates the line shown.

(legend continued on next page)

expression of signaling pathway effectors, thereby leading to variability in the capacity of differentiation.

## DISCUSSION

In this study, we established that naive mESC lines derived from mouse strains with different genetic backgrounds display variable differentiation capacities even in identical culture conditions. Importantly, mESC lines independently derived from the same genetic background displayed a similar capacity of differentiation, thereby excluding the possibility that experimental variations could explain the divergence among pluripotent cell lines. Thus, the variability commonly observed in hPSCs could be shared among different species independent of the pluripotent state or culture conditions. Importantly, mouse strains are genetically well defined, and the genetic differences among them are considerable (Keane et al., 2011; Lilue et al., 2018). They therefore provide a unique model system to investigate the impact of genetics on phenotypic variability.

Importantly, our results show that divergence between mESC lines starts during the transition from the naive state to the epiblast-like state. Thus, variability in the capacity of differentiation can also originate directly from the naive state and not from subsequent pluripotent states. Our study also uncovered that the Wnt pathway plays an important role in this transition, and the activity of this signaling fluctuates among mESC lines of different genetic backgrounds. This variation is likely to have a major impact on cell fate decisions, as Wnt signaling is a key regulator of naive pluripotency, germ-layer specification (Sato et al., 2004), and primitive endoderm differentiation (Anderson et al., 2017). Interestingly, variations in Wnt signaling have also been shown to influence the differentiation behavior of human PSCs (Blauwkamp et al., 2012; Strano et al., 2020). In agreement with this, we observed that naive hESCs also display different levels of Wnt signaling. Thus, mechanisms affecting the activity of this pathway are likely to have a major impact on pluripotent stem cells across species. Nonetheless, modulating Wnt pathway activity using small molecules did not completely erase differences among naive mESC lines. Thus, complex combinations of factors and signaling are likely to influence phenotypic variability among pluripotent stem cell lines, and further functional analyses are required to fully uncover these interactions.

Finally, our results imply that genetic mechanisms can influence the activity of signaling pathways such as Wnt. We identified potential distal expression QTL regulating Wnt pathway genes such as Axin2 and Lef1. Thus, genetic variants could affect cell fate decisions by influencing extra-cellular signaling inputs. However, these analyses also show that phenotypic variability is likely to be caused by a diversity of genetic variants. Accordingly, we found eQTL in genes controlling FGF signaling, thereby uncovering potential links with yet another major signaling pathway influencing cell fate decisions. Phenotypic variability is likely to be induced by a combination of genetic variants that individually may have limited impact, and direct functional validations are likely to yield limited information. Nonetheless, our study highlights the utility of a systems genetics approach using the Diversity Outbred mouse resource to link cellular phenotypes with specific genetic variants and identify targets for further studies of the underlying molecular mechanisms. This approach is further exemplified by Skelly et al. (2020), who have exploited molecular profiling of a large cohort of Diversity Outbred mESC lines to expose genetic mechanisms controlling metastability of the naive pluripotent state.

To conclude, our results are conceptually important, as they suggest that genetic variations could have a major impact on developmental processes. Indeed, fine modulation of signaling pathways could diverge among genetically diverse mouse strains, resulting in phenotypic variability. This phenomenon would be particularly acute *in vitro*, where the same culture conditions are systematically applied, while *in vivo* compensation mechanisms and precise fine-tuning of extra-cellular cues will allow normal development. Nonetheless, such variability is likely to have a major influence on the mechanisms underpinning cell fate decision and at later stages on disease onset (Doetschman, 2009). Our study also has major implications for hPSCs. Indeed, our results indicate that variability among lines is intrinsic to their genetic background and that improving methods of derivation, reprogramming, and culture conditions is unlikely to solve this issue in its entirety. Ultimately, methods of differentiation might need to be optimized for each cell line, and understanding the detailed molecular mechanisms orchestrating differentiation remains the best solution to developing improved methods for the production of clinically relevant cells.

### Limitations of Study

The aim of our study was to focus on the impact of genetic background on variability of ground-state pluripotent stem cells while keeping all other parameters, such as method of derivation and conditions for culture and differentiation, constant among all lines. This does not mean that these factors cannot contribute to variability in other contexts.

Our findings imply that epigenetic resetting associated with the naive pluripotent state is not sufficient to overcome the impact of genetic background. Nonetheless, we cannot exclude that epigenetics could also play a role. As an example, previous studies have shown that major epigenetic processes such as X

(C) Luciferase assay for TOP FLASH promotor activity after 24 h in the indicated culture conditions. Data are normalized to the CAST genetic background and are the mean from two independent experiments for each genetic background, two lines per background. Error bars represent SD of the means. $^{**}p < 0.005$ and $^{***}p < 0.0005$ (two-tailed t test).

(D) Gene expression analysis of selected Wnt signaling target genes after 2 days in the indicated culture conditions. Data shown are the mean from two lines per genetic background. Error bars represent SD of the means. $^{*}p < 0.05$ and $^{**}p < 0.005$ (two-tailed t test).

(E) Quantification of flow cytometry for OTX2 after 2 days in the indicated culture conditions. Data shown are the mean from two lines per genetic background. Error bars represent SD of the means. $^{*}p < 0.05$ (two-tailed t test).

(F) eQTL analysis for Axin2 showing two LOD score peaks on chromosome 3 and 5.

(G) Founder haplotype analysis of CAST, B6, and C3H genetic backgrounds. Details of the analysis can be found in STAR Methods.

(H) eQTL analysis for Fgfr1l expression showing a strong *cis* eQTL on chromosome 5 LOD > 14.

CellPress
OPEN ACCESS

inactivation could influence the differentiation capacity of female hPSCs (Anguera et al., 2012; Shen et al., 2008). For this reason, we focused our study on male mESC lines, which can contribute efficiently to chimeras after blastocyst injection. Performing similar studies on female mESCs from different genetic backgrounds could reveal specific variations associated with X-related epigenetic regulation.

Finally, the genetic differences among mouse strains are greater than among human individuals; therefore the results of this study cannot be directly extrapolated but can serve as the basis of similar investigations in the human system.

## STAR★METHODS

Detailed methods are provided in the online version of this paper and include the following:

- KEY RESOURCES TABLE
- RESOURCE AVAILABILITY
  - Lead Contact
  - Materials Availability
  - Data and Code Availability
- EXPERIMENTAL MODEL AND SUBJECT DETAILS
  - Cell Lines
- METHOD DETAILS
  - Cell Line Differentiation
  - Colony Forming Assay
  - Flow Cytometry
  - Cell cycle analysis
  - Quantitative PCR
  - Cellular Fractionation
  - Western Blot
  - Immunocytochemistry
  - Luciferase Reporter Assay
  - ELISA Assay
  - Bulk RNA-seq on inbred strain mESCs
  - Expression QTL Mapping
  - Single Cell analysis
  - Demultiplexing of 10x Data
  - Analysis of 10x Data
- QUANTIFICATION AND STATISTICAL ANALYSIS

## SUPPLEMENTAL INFORMATION

Supplemental Information can be found online at https://doi.org/10.1016/j.stem.2020.07.019.

## ACKNOWLEDGMENTS

This work was supported by the European Research Council Grant New-Chol (L.V., A.O., R.A.T.), the INTENS EuFP8 grant (D.O.), a British Heart Foundation (BHF) PhD Studentship as part of the BHF Oxbridge Centre of Regenerative Medicine (G.A.C.), a Gates PhD scholarship (B.T.W.), NIH grant P40 OD011102 (L.G.R.), the Cambridge Hospitals National Institute for Health Research Biomedical Research Center (L.V., S.B.), and a core support grant from the Wellcome Trust and Medical Research Council to the Wellcome Trust – Medical Research Council Cambridge Stem Cell Institute.

## AUTHOR CONTRIBUTIONS

Conceptualization, D.O., S.B., and L.V.; Methodology, D.O., S.B., R.A.T., and L.V.; Investigation, D.O., S.B., G.A.C., A.C., P.J.R.-G., and A.O.; Formal Analysis, D.O., S.B., G.A.C., A.C., D.M., S.A., A.O., and D.A.S.; Writing – Original Draft, D.O., S.B., and L.V.; Writing – Review & Editing, D.O., S.B., A.C., S.A., R.A.T., B.T.W., A.O., D.A.S., C.L.B., P.J.R.-G., S.C.M., L.G.R., and L.V.; Funding Acquisition, L.V.; Resources, L.V., T.C., G.A.C., C.L.B., P.J.R.-G., S.C.M., and L.G.R.; Supervision, S.C.M., O.S., L.G.R., and L.V.

## DECLARATION OF INTERESTS

The authors declare no competing interests.

Received: February 23, 2019
Revised: April 10, 2020
Accepted: July 24, 2020
Published: August 13, 2020

## REFERENCES

Acampora, D., Di Giovannantonio, L.G., and Simeone, A. (2013). Otx2 is an intrinsic determinant of the embryonic stem cell state and is required for transition to a stable epiblast stem cell condition. Development *140*, 43–55.

Alasoo, K., Rodrigues, J., Mukhopadhyay, S., Knights, A.J., Mann, A.L., Kundu, K., Hale, C., Dougan, G., and Gaffney, D.J.; HIPSCI Consortium (2018). Shared genetic effects on chromatin and gene expression indicate a role for enhancer priming in immune response. Nat. Genet. *50*, 424–431.

Alexa, A., and Rahnenführer, J. (2019). Gene set enrichment analysis with topGO. https://bioconductor.org/packages/release/bioc/vignettes/topGO/inst/doc/topGO.pdf.

Anderson, K.G.V., Hamilton, W.B., Roske, F.V., Azad, A., Knudsen, T.E., Canham, M.A., Forrester, L.M., and Brickman, J.M. (2017). Insulin fine-tunes self-renewal pathways governing naive pluripotency and extra-embryonic endoderm. Nat. Cell Biol. *19*, 1164–1177.

Anguera, M.C., Sadreyev, R., Zhang, Z., Szanto, A., Payer, B., Sheridan, S.D., Kwok, S., Haggarty, S.J., Sur, M., Alvarez, J., et al. (2012). Molecular signatures of human induced pluripotent stem cells highlight sex differences and cancer genes. Cell Stem Cell *11*, 75–90.

Bernemann, C., Greber, B., Ko, K., Sterneckert, J., Han, D.W., Araúzo-Bravo, M.J., and Schöler, H.R. (2011). Distinct developmental ground states of epiblast stem cell lines determine different pluripotency features. Stem Cells *29*, 1496–1503.

Blauwkamp, T.A., Nigam, S., Ardehali, R., Weissman, I.L., and Nusse, R. (2012). Endogenous Wnt signalling in human embryonic stem cells generates an equilibrium of distinct lineage-specified progenitors. Nat. Commun. *3*, 1070.

Bock, C., Kiskinis, E., Verstappen, G., Gu, H., Boulting, G., Smith, Z.D., Ziller, M., Croft, G.F., Amoroso, M.W., Oakley, D.H., et al. (2011). Reference maps of human ES and iPS cell variation enable high-throughput characterization of pluripotent cell lines. Cell *144*, 439–452.

Boulting, G.L., Kiskinis, E., Croft, G.F., Amoroso, M.W., Oakley, D.H., Wainger, B.J., Williams, D.J., Kahler, D.J., Yamaki, M., Davidow, L., et al. (2011). A functionally characterized test set of human induced pluripotent stem cells. Nat. Biotechnol. *29*, 279–286.

Broman, K.W., Sen, S., Owens, S.E., Manichaikul, A., Southard-Smith, E.M., and Churchill, G.A. (2006). The X chromosome in quantitative trait locus mapping. Genetics *174*, 2151–2158.

Brons, I.G.M., Smithers, L.E., Trotter, M.W.B., Rugg-Gunn, P., Sun, B., Chuva de Sousa Lopes, S.M., Howlett, S.K., Clarkson, A., Ahrlund-Richter, L., Pedersen, R.A., and Vallier, L. (2007). Derivation of pluripotent epiblast stem cells from mammalian embryos. Nature *448*, 191–195.

Buecker, C., Srinivasan, R., Wu, Z., Calo, E., Acampora, D., Faial, T., Simeone, A., Tan, M., Swigut, T., and Wysocka, J. (2014). Reorganization of enhancer patterns in transition from naive to primed pluripotency. Cell Stem Cell *14*, 838–853.

Butcher, L.M., Ito, M., Brimpari, M., Morris, T.J., Soares, F.A.C., Ährlund-Richter, L., Carey, N., Vallier, L., Ferguson-Smith, A.C., and Beck, S. (2016). Non-CG DNA methylation is a biomarker for assessing endodermal differentiation capacity in pluripotent stem cells. Nat. Commun. *7*, 10458.

Carcamo-Orive, I., Hoffman, G.E., Cundiff, P., Beckmann, N.D., D'Souza, S.L., Knowles, J.W., Patel, A., Papatsenko, D., Abbasi, F., Reaven, G.M., et al. (2017). Analysis of transcriptional variability in a large human iPSC library reveals genetic and non-genetic determinants of heterogeneity. Cell Stem Cell *20*, 518–532.e9.

Churchill, G.A., Airey, D.C., Allayee, H., Angel, J.M., Attie, A.D., Beatty, J., Beavis, W.D., Belknap, J.K., Bennett, B., Berrettini, W., et al.; Complex Trait Consortium (2004). The Collaborative Cross, a community resource for the genetic analysis of complex traits. Nat. Genet. *36*, 1133–1137.

Cuomo, A.S.E., Seaton, D.D., McCarthy, D.J., Martinez, I., Bonder, M.J., Garcia-Bernardo, J., Amatya, S., Madrigal, P., Isaacson, A., Buettner, F., et al. (2020). Single-cell RNA-sequencing of differentiating iPS cells reveals dynamic genetic effects on gene expression. Nat. Commun. *11*, 1–14.

Czechanski, A., Byers, C., Greenstein, I., Schrode, N., Donahue, L.R., Hadjantonakis, A.-K., and Reinholdt, L.G. (2014). Derivation and characterization of mouse embryonic stem cells from permissive and nonpermissive strains. Nat. Protoc. *9*, 559–574.

DeBoever, C., Li, H., Jakubosky, D., Benaglio, P., Reyna, J., Olson, K.M., Huang, H., Biggs, W., Sandoval, E., D'Antonio, M., et al. (2017). Large-scale profiling reveals the influence of genetic variation on gene expression in human induced pluripotent stem cells. Cell Stem Cell *20*, 533–546.e7.

Ditadi, A., Sturgeon, C.M., Tober, J., Awong, G., Kennedy, M., Yzaguirre, A.D., Azzola, L., Ng, E.S., Stanley, E.G., French, D.L., et al. (2015). Human definitive haemogenic endothelium and arterial vascular endothelium represent distinct lineages. Nat. Cell Biol. *17*, 580–591.

Doetschman, T. (2009). Influence of genetic background on genetically engineered mouse phenotypes. Methods Mol. Biol. *530*, 423–433.

Gafni, O., Weinberger, L., Mansour, A.A., Manor, Y.S., Chomsky, E., Ben-Yosef, D., Kalma, Y., Viukov, S., Maza, I., Zviran, A., et al. (2013). Derivation of novel human ground state naive pluripotent stem cells. Nature *504*, 282–286.

Gatti, D.M., Svenson, K.L., Shabalin, A., Wu, L.Y., Valdar, W., Simecek, P., Goodwin, N., Cheng, R., Pomp, D., Palmer, A., et al. (2014). Quantitative trait locus mapping methods for diversity outbred mice. G3 (Bethesda) *4*, 1623–1633.

Han, D.W., Tapia, N., Joo, J.Y., Greber, B., Araúzo-Bravo, M.J., Bernemann, C., Ko, K., Wu, G., Stehling, M., Do, J.T., and Schöler, H.R. (2010). Epiblast stem cell subpopulations represent mouse embryos of distinct pregastrulation stages. Cell *143*, 617–627.

Huang, Y., McCarthy, D.J., and Stegle, O. (2019). Vireo: Bayesian demultiplexing of pooled single-cell RNA-seq data without genotype reference. Genome Biol. *20*, 273.

Keane, T.M., Goodstadt, L., Danecek, P., White, M.A., Wong, K., Yalcin, B., Heger, A., Agam, A., Slater, G., Goodson, M., et al. (2011). Mouse genomic variation and its effect on phenotypes and gene regulation. Nature *477*, 289–294.

Kilpinen, H., Goncalves, A., Leha, A., Afzal, V., Alasoo, K., Ashford, S., Bala, S., Bensaddek, D., Casale, F.P., Culley, O.J., et al. (2017). Common genetic variation drives molecular heterogeneity in human iPSCs. Nature *546*, 370–375.

Langmead, B., Trapnell, C., Pop, M., and Salzberg, S.L. (2009). Ultrafast and memory-efficient alignment of short DNA sequences to the human genome. Genome Biol. *10*, R25.

Leek, J.T., Johnson, W.E., Parker, H.S., Jaffe, A.E., and Storey, J.D. (2012). SVA detailed instruction. Bioinformatics *28*, 882–883.

Leitch, H.G., McEwen, K.R., Turp, A., Encheva, V., Carroll, T., Grabole, N., Mansfield, W., Nashun, B., Knezovich, J.G., Smith, A., et al. (2013). Naive pluripotency is associated with global DNA hypomethylation. Nat. Struct. Mol. Biol. *20*, 311–316.

Li, H., and Durbin, R. (2009). Fast and accurate short read alignment with Burrows-Wheeler transform. Bioinformatics *25*, 1754–1760.

Liberzon, A., Birger, C., Thorvaldsdottir, H., Ghandi, M., Mesirov, J.P., and Tamayo, P. (2015). The Molecular Signatures Database (MSigDB) hallmark gene set collection. Cell Syst *1*, 417–425.

Lilue, J., Doran, A.G., Fiddes, I.T., Abrudan, M., Armstrong, J., Bennett, R., Chow, W., Collins, J., Collins, S., Czechanski, A., et al. (2018). Sixteen diverse laboratory mouse reference genomes define strain-specific haplotypes and novel functional loci. Nat. Genet. *50*, 1574–1583.

Love, M.I., Huber, W., and Anders, S. (2014). Moderated estimation of fold change and dispersion for RNA-seq data with DESeq2. Genome Biol. *15*, 550.

Marks, H., Kalkan, T., Menafra, R., Denissov, S., Jones, K., Hofemeister, H., Nichols, J., Kranz, A., Stewart, A.F., Smith, A., and Stunnenberg, H.G. (2012). The transcriptional and epigenomic foundations of ground state pluripotency. Cell *149*, 590–604.

Nazor, K.L., Altun, G., Lynch, C., Tran, H., Harness, J.V., Slavin, I., Garitaonandia, I., Müller, F.-J., Wang, Y.-C., Boscolo, F.S., et al. (2012). Recurrent variations in DNA methylation in human pluripotent stem cells and their differentiated derivatives. Cell Stem Cell *10*, 620–634.

Nichols, J., and Smith, A. (2009). Naive and primed pluripotent states. Cell Stem Cell *4*, 487–492.

Nishizawa, M., Chonabayashi, K., Nomura, M., Tanaka, A., Nakamura, M., Inagaki, A., Nishikawa, M., Takei, I., Oishi, A., Tanabe, K., et al. (2016). Epigenetic variation between human induced pluripotent stem cell lines is an indicator of differentiation capacity. Cell Stem Cell *19*, 341–354.

Osafune, K., Caron, L., Borowiak, M., Martinez, R.J., Fitz-Gerald, C.S., Sato, Y., Cowan, C.A., Chien, K.R., and Melton, D.A. (2008). Marked differences in differentiation propensity among human embryonic stem cell lines. Nat. Biotechnol. *26*, 313–315.

Raghupathy, N., Choi, K., Vincent, M.J., Beane, G.L., Sheppard, K.S., Munger, S.C., Korstanje, R., Pardo-Manual de Villena, F., and Churchill, G.A. (2018). Hierarchical analysis of RNA-seq reads improves the accuracy of allele-specific expression. Bioinformatics *34*, 2177–2184.

Rostovskaya, M., Stirparo, G.G., and Smith, A. (2019). Capacitation of human naïve pluripotent stem cells for multi-lineage differentiation. Development *146*. https://dev.biologists.org/content/146/7/dev172916.

Rouhani, F., Kumasaka, N., de Brito, M.C., Bradley, A., Vallier, L., and Gaffney, D. (2014). Genetic background drives transcriptional variation in human induced pluripotent stem cells. PLoS Genet. *10*, e1004432.

Sato, N., Meijer, L., Skaltsounis, L., Greengard, P., and Brivanlou, A.H. (2004). Maintenance of pluripotency in human and mouse embryonic stem cells through activation of Wnt signaling by a pharmacological GSK-3-specific inhibitor. Nat. Med. *10*, 55–63.

Shen, Y., Matsuno, Y., Fouse, S.D., Rao, N., Root, S., Xu, R., Pellegrini, M., Riggs, A.D., and Fan, G. (2008). X-inactivation in female human embryonic stem cells is in a nonrandom pattern and prone to epigenetic alterations. Proc. Natl. Acad. Sci. U S A *105*, 4709–4714.

Skelly, D.A., Czechanski, A., Byers, C., Aydin, S., Spruce, C., Olivier, C., Choi, K., Gatti, D.M., Raghupathy, N., Keele, G.R., et al. (2020). Mapping the effects of genetic variation on chromatin state and gene expression reveals loci that control ground state pluripotency. Cell Stem Cell *27*. Published online August 13, 2020. https://doi.org/10.1016/j.stem.2020.07.019.

Sleeman, M., Fraser, J., McDonald, M., Yuan, S., White, D., Grandison, P., Kumble, K., Watson, J.D., and Murison, J.G. (2001). Identification of a new fibroblast growth factor receptor, FGFR5. Gene *271*, 171–182.

Smith, A. (2017). Formative pluripotency: the executive phase in a developmental continuum. Development *144*, 365–373.

Strano, A., Tuck, E., Stubbs, V.E., and Livesey, F.J. (2020). Variable outcomes in neural differentiation of human PSCs arise from intrinsic differences in developmental signaling pathways. Cell Rep. *31*, 107732.

Sugimoto, M., Kondo, M., Koga, Y., Shiura, H., Ikeda, R., Hirose, M., Ogura, A., Murakami, A., Yoshiki, A., Chuva de Sousa Lopes, S.M., and Abe, K. (2015). A simple and robust method for establishing homogeneous mouse epiblast stem cell lines by wnt inhibition. Stem Cell Reports *4*, 744–757.

Takashima, Y., Guo, G., Loos, R., Nichols, J., Ficz, G., Krueger, F., Oxley, D., Santos, F., Clarke, J., Mansfield, W., et al. (2014). Resetting transcription factor control circuitry toward ground-state pluripotency in human. Cell *158*, 1254–1269.

CellPress
OPEN ACCESS

ten Berge, D., Kurek, D., Blauwkamp, T., Koole, W., Maas, A., Eroglu, E., Siu, R.K., and Nusse, R. (2011). Embryonic stem cells require Wnt proteins to prevent differentiation to epiblast stem cells. Nat. Cell Biol. *13*, 1070–1075.

Theunissen, T.W., Powell, B.E., Wang, H., Mitalipova, M., Faddah, D.A., Reddy, J., Fan, Z.P., Maetzel, D., Ganz, K., Shi, L., et al. (2014). Systematic identification of culture conditions for induction and maintenance of naive human pluripotency. Cell Stem Cell *15*, 471–487.

Tropepe, V., Hitoshi, S., Sirard, C., Mak, T.W., Rossant, J., and van der Kooy, D. (2001). Direct neural fate specification from embryonic stem cells: a primitive mammalian neural stem cell stage acquired through a default mechanism. Neuron *30*, 65–78.

Veeman, M.T., Slusarski, D.C., Kaykas, A., Louie, S.H., and Moon, R.T. (2003). Zebrafish prickle, a modulator of noncanonical Wnt/Fz signaling, regulates gastrulation movements. CurrBiol *13*, 680–685.

Wolf, F.A., Angerer, P., and Theis, F.J. (2018). SCANPY: large-scale single-cell gene expression data analysis. Genome Biol. *19*, 15.

Yang, S.-H., Kalkan, T., Morissroe, C., Marks, H., Stunnenberg, H., Smith, A., and Sharrocks, A.D. (2014). Otx2 and Oct4 drive early enhancer activation during embryonic stem cell transition from naive pluripotency. Cell Rep. *7*, 1968–1981.

Ying, Q.-L., Wray, J., Nichols, J., Batlle-Morera, L., Doble, B., Woodgett, J., Cohen, P., and Smith, A. (2008). The ground state of embryonic stem cell self-renewal. Nature *453*, 519–523.

Zhao, T., Fu, Y., Zhu, J., Liu, Y., Zhang, Q., Yi, Z., Chen, S., Jiao, Z., Xu, X., Xu, J., et al. (2018). Single-cell RNA-seq reveals dynamic early embryonic-like programs during chemical reprogramming. Cell Stem Cell *23*, 31–45.e7.

## STAR★METHODS

### KEY RESOURCES TABLE

| REAGENT or RESOURCE | SOURCE | IDENTIFIER |
|---|---|---|
| Antibodies | | |
| Mouse monoclonal anti-Oct4 | Santa Cruz | Cat#SC-5279; RRID: AB_628051 |
| Goat polyclonal anti-Otx2 | R & D | Cat#AF1979; RRID: AB_2157172 |
| Goat polyclonal anti-Sox1 | R & D | Cat#AF3369; RRID: AB_2239879 |
| Goat polyclonal anti-Sox17 | R & D | Cat#AF1924; RRID: AB_355060 |
| Mouse monoclonal anti-β Catenin (Active form) | Merck-Millipore | Cat#05-665; RRID: AB_309887 |
| Goat polyclonal anti-β Catenin (Total) | R & D | Cat#AF1329; RRID: AB_354736 |
| Mouse monoclonal anti-α Tubulin | Sigma | Cat#T6199; RRID: AB_477583 |
| Rabbit polyclonal anti-Histone H3 | Sigma | Cat#H0164; RRID: AB_532248 |
| Goat polyclonal anti-Nanog (human) | R & D | Cat#AF1997; RRID: AB_355097 |
| Biological Samples | | |
| Naive human cell lysates | Dr Peter Rugg-Gunn | N/A |
| Chemicals, Peptides, and Recombinant Proteins | | |
| ActivinA | Dr Marko Hyvönen, Cambridge University | N/A |
| FGF2 | Dr Marko Hyvönen, Cambridge University | N/A |
| Ms LIF | Dr Marko Hyvönen, Cambridge University | N/A |
| BMP4 | R & D | Cat#314-BP |
| CHIR99021 | Tocris Bioscience | Cat#4423/10 |
| PD0325901 | Tocris Bioscience | Cat#4192 |
| LY294002 | Promega | Cat#V1201 |
| Retinoic Acid | Sigma | R2625 |
| IWP2 | Tocris | Cat# 3533/10 |
| Critical Commercial Assays | | |
| Kapa Sybr Fast Low Rox | Sigma | Cat#KK4622 |
| Dual Luciferase Reporter Assay | Promega | Cat#E1910 |
| Total β Catenin ELISA kit | Life Technologies | Cat#KHO1211 |
| GenElute Total Mammalian RNA Extraction Kit | Sigma | Cat#RTN350 |
| Click-iT EdU Pacific Blue Flow Cytometry Assay Kit | ThermoFisher | Cat#C10418 |
| Deposited Data | | |
| Raw data | This paper | http://dx.doi.org/10.17632/gy6t9rv8jd.1 |
| Single Cell RNA Seq Raw data | This paper | ArrayExpress: E-MTAB-8844 |
| Mouse Embryonic Fibroblast (MEF) scRNA Seq data | Zhao et al., 2018 | BIG: CRA000932; GEO: GSE114952 |
| Bulk RNA Seq data | Skelly et al., 2020 | ArrayExpress: E-MTAB-7730, E-MTAB-7728 (founder inbred strain mESC RNA-Seq and diversity outcross mESC RNA-Seq) |
| Mouse strain SNP datasets | Wellcome Trust Sanger Institute | ftp://ftp-mouse.sanger.ac.uk/current_snps/mgp.v5.merged.snps_all.dbSNP142.vcf.gz |
| Mouse genome dataset C57BL/6NJ | Keane et al., 2011 | EMBL-EBI: ERP000041 |
| Mouse genome dataset C3H/HeJ | Keane et al., 2011 | EMBL-EBI: ERP000040 |

(Continued on next page)

CellPress
OPEN ACCESS

*Continued*

| REAGENT or RESOURCE | SOURCE | IDENTIFIER |
|---|---|---|
| Mouse genome dataset CAST/EiJ | Keane et al., 2011 | EMBL-EBI: ERP000042 |
| The Molecular Signature Database Hallmark Gene Set Collection (MSigDB) | Liberzon et al., 2015 | https://www.gsea-msigdb.org/gsea/msigdb/genesets.jsp?collection=H |
| Experimental Models: Cell Lines | | |
| Mouse: C57BL/6J | Jackson Laboratory | Strain ID JR#000664; RRID: CVCL_ZL17 |
| Mouse: C3H/HeJ | Jackson Laboratory | Strain ID JR#000659; RRID: CVCL_2H67 |
| Mouse: CAST/EiJ | Jackson Laboratory | Strain ID JR#000928; RRID: CVCL_ZL18 |
| Mouse: PWD/PhJ | Jackson Laboratory | Strain ID JR#004660; RRID: CVCL_2H68 |
| Oligonucleotides | | |
| See Primers Table for Oligonucleotide sequences used in this study | | N/A |
| Recombinant DNA | | |
| M50 Super 8x TOPFlash | Veeman et al., 2003 | Addgene plasmid#12456; RRID: Addgene_12456 |
| pGL4.74[*hRluc*/TK] vector | Promega | Cat#E6931 |
| Software and Algorithms | | |
| ImageJ | https://imagej.net/Welcome | RRID:SCR_003070 |
| Python | Python Software Foundation | https://www.python.org ; RRID: SCR_008394 |
| Scanpy (1.4.4.post1) | | https://scanpy.readthedocs.io/en/stable/index.html ; RRID:SCR_018139 |
| topGO | Alexa and Rahnenführer, 2019 | https://bioconductor.org/packages/release/bioc/html/topGO.html ; RRID:SCR_014798 |

## RESOURCE AVAILABILITY

### Lead Contact

Further information and requests for resources and reagents should be directed to and will be fulfilled by the Lead Contact, Ludovic Vallier (lv225@cam.ac.uk).

### Materials Availability

This study did not generate new unique reagents.

### Data and Code Availability

The accession number for the Single Cell RNA-Seq data reported in this paper is ArrayExpress: E-MTAB-8844.

## EXPERIMENTAL MODEL AND SUBJECT DETAILS

### Cell Lines

Mouse embryonic stem cell (mESC) lines were derived from different blastocysts of inbred strains (CAST/EiJ (CAST); RRID: CVCL_ZL18, C57BL/6J (B6); RRID:CVCL_ZL17, C3H/HeJ (C3H); RRID: CVCL_2H67 and PWD/PhJ (PWD); RRID: CVCL_2H68). All lines used were male and were obtained from the Jackson Laboratory.

Euploid (> 70%), germline competent, male mESCs were derived from B6: C57BL/6J (The Jackson Laboratory strain ID JR#000664), CAST: CAST/EiJ (JR#000928), PWD: PWD/PhJ (JR#004660), 129S1/SvImJ (JR#002448), C3H: C3H/HeJ (JR#000659) as previously described (Czechanski et al., 2014). Briefly, embryos from natural matings were collected at 3.5 days post coitum by flushing uteri with M2 medium (Millipore). Blastocysts were transferred directly to MEF (mouse embryonic fibroblast, irradiated) feeder layers in 4-well IVF (*in vitro* fertilization) culture dishes, 1–2 blastocysts per well. Any flushed morulae were cultured overnight in KSOM/M16 (Millipore) medium and the resulting blastocysts were transferred to MEF feeder layers the following day. Blastocysts were then cultured at 37°C, 5% CO2 for 8–10 days in 2i medium (1:1 mixture of DMEM-F12/N2 (DMEM-F12 supplemented with N-2) and Neurobasal/B27 (Neurobasal supplemented with B27) with 1 × (vol/vol) penicillin-streptomycin, 1 mM GlutaMAX, 0.5 mM sodium pyruvate, 0.1 mM MEM NEAA, 0.1 mM 2-mercaptoethanol, 103 IU of LIF, 1 μM PD0325901 and 3 μM CHIR99021). During the first 4 days, blastocysts were undisturbed to allow for zona hatching and attachment of the embryos to

the feeder layers. During days 5–10, the media were replaced every other day and inner cell mass (ICM) outgrowth was observed. Outgrowths were then mechanically disaggregated and cultured in the presence of feeders in ESM +2i (FBS ES medium is DMEM-high glucose supplemented with 15% (vol/vol) FBS, 1 × penicillin-streptomycin, 2 mM GlutaMAX, 1 mM sodium pyruvate, 0.1 mM MEM NEAA, 0.1 mM 2-mercaptoethanol, 103 IU of LIF, 1 μM PD0325901 and 3 μM CHIR99021). Up to 20 cell lines per strain were expanded and a minimum of three male cell lines were selected for further study as follows: cells were tested for cell culture contaminants through PCR and visible examination, euploidy through spreading and counting of mitotic chromosomes, and germline competence through microinjection of mESCs into host embryo; resulting chimeras were scored on the basis of coat color contribution and male chimeras were tested for germline transmission of the mESC genome (by assessing coat color).

Lines were thawed onto CF1 irradiated mouse embryonic fibroblasts (Life Technologies, A34181) in KSR medium (Advanced DMEM/F12 (80%), knock-out serum replacer (20%, GIBCO), L-Glutamine (1mM), 2-Mercaptoethanol (0.1mM) and Penicillin/Streptomycin (1%)), supplemented with 10ng/ml LIF. Cells were then transferred to 2iL culture by splitting with TrypLE and plating around 2-3 x$10^4$ cells/$cm^2$ on gelatine coated plates in 2iL medium. They were grown for a minimum of 3 passages before the start of experiments to ensure that the cells are sufficiently adapted to culture in 2iL. Cells were passaged every two days by dissociation with TrypLE and 2-3 x$10^4$ cells/$cm^2$ were plated on gelatine coated plates (0.1%–0.2% porcine gelatin in PBS for at least 20 minutes at room temperature) in N2B27 medium (1:1 mix of Neurobasal medium and DMEM/F12, with 1:50 B27 and 1:100 N2 supplements, 2mM Glutamax, 50μM β-Mercaptoethanol, 50μg/ml Penicillin-streptomycin) supplemented with 10ng/ml LIF, 3 μM CHIR99021 and 1 μM PD0325901. Cells were grown in a humidified incubator at 37°C and 5% CO2.

## METHOD DETAILS

### Cell Line Differentiation

mESCs were dissociated with TrypLE and seeded at a density of 5-15 × $10^3$ cells per $cm^2$ in 2iL medium (see above for composition). After 24 hours the cells were washed once in D-PBS and then treated with the different growth conditions specified below. All cell lines were differentiated simultaneously using cells of the same passage number and were repeated at least twice.

For unguided differentiation, cells were grown in N2B27 medium (see above for composition) without the addition of supplements.

For endoderm differentiation, cells were grown for 48 hours in N2B27 medium supplemented with 20ng/mL ActivinA and 20ng/mL FGF2. For the following 24 hours the medium was changed to CDM-PVA (IMDM:F-12 (1:1), Conc. Lipids 1%, 1-thioglycerol 450mM, Insulin 7ug/mL, Transferrin 15ug/mL, Penicillin-streptomycin 1%, PVA 1mg/mL) supplemented with 20ng/mL FGF2, 100ng/mL ActivinA, 10 μM LY294002 (Promega), 10ng/mL BMP4 and 3 μM CHIR99021. The medium was then changed to the same composition without CHIR99021 for a further 48 hours. The following two days, the cells were cultured in RPMI, B27 (1:50, Life Technologies 17504-044), NEAA (1:100, Life Technologies 11140-035) supplemented with 80ng/mL FGF2, 100ng/mL ActivinA.

For primitive endoderm differentiation, cells were grown in RPMI1640 plus Glutamax (Life Technologies 61870-044) supplemented with 1 μM Retinoic Acid (Sigma R2625), 3 μM CHIR99201, 20ng/ml Activin and 10ng/ml LIF. The medium was changed daily for 12 days.

For cardiac differentiation, mESCs were split as described above and then seeded into non-coated dishes (special low-attachment dishes can be used if necessary) in KSR medium (see above for composition details) to allow the formation of embryoid bodies for two days. Sporadically attaching aggregates can be gently washed off the plate by pipetting. After that, KSR medium was supplemented with ActivinA (20ng/ml) and BMP4 (20ng/ml) for the subsequent 4 days and EBs were allowed to attach to the plate. The media was changed every two days thereafter to KSR medium without additional supplements. Spontaneous contractions appeared in the culture after about 12 days of differentiation. On differentiation day 20, cells were lysed and analyzed for expression of cardiac markers by qPCR.

For hematopoetic differentiation, cells were dissociated into small clumps (size of about 20 cells per clump) by 5-10 minutes incubation with 50 uM EDTA and scraping, and resuspended as embryoid bodies in serum-free differentiation (SFD) medium (5% IMDM, 25% Ham's F12, 1% N2 (Life technologies 17502001), 0.5% B27, 0.05% BSA, 1mM Ascorbic acid, 4.5 × $10^{-4}$M Monothioglycerol, 2mM L-Glutamine, 150ug/mL Transferrin (Sigma T1147), 10ng/mL Penicillin/Streptomycin). At Day 1 the medium was changed to SFD supplemented with 10ng/mL BMP4, 10ng/mL FGF2 and 10mg/mL ActivinA. At Day 2.5 the medium was changed to SFD supplemented with 10ng/mL FGF2, 15ng/mL VEGF (Peprotech 100-20-100), 10ng/mL IL6(R&D 206-IL-010) and 5ng/mL IL11 (R&D 218-IL-005). At Day 4 the medium was changed to SFD as on Day 2.5 with the addition of 50ng/mL SCF (R&D 255-SC-200), 5ng/mL IGF1(Peprotech 100-11) and 2U/mL EPO (Peprotech 100-64).The same media composition was maintained on Day 5 and the cells were collected on Day 6 for further analysis.

### Colony Forming Assay

The assay was performed using the MethoCult H4435 Enriched medium (STEMCELL Technologies), following the manufacturer's instructions. Haematopoetic differentiated cells were dissociated into single cells by 5 minutes incubation with TrypLE, counted, and 5000 cells/line were added to 1.2 mL of MethoCult media, mixed by vortexing and plated on non-treated 35 mm culture dishes (Corning) and incubated for 14 days at 37°C, 5% CO2 and 5% O2. Different types of colony were scored based on morphology, following the manufacturer's instructions.

### Flow Cytometry

Cells were dissociated to single cells with TrypLE and fixed in 1x Fix/Perm buffer (BD Bioscience 554722) for 20 minutes at 4°C. Cells were washed once with D-PBS and then blocked for 30 minutes in Perm/Wash (BD Bioscience 554723). Cells were then incubated with primary antibody (1:300 OCT4 Santa Cruz SC-5279, RRID: AB_628051, 1:300 OTX2 R&D AF1979, RRID: AB_2157172, 1:300 SOX1 R&D AF3369, RRID: AB_2239879, 1:300 SOX17 R&D AF1924, RRID: AB_355060) in Perm/Wash at room temperature for 3 hours. After three washes with Perm/Wash, cells were incubated with the appropriate secondary antibody (1:300) in Perm/Wash for 1 hour at room temperature. Cells were washed 3 times with Perm/Wash, resuspended in PBS and run on a BD FACSCantoII flow cytometer. Data was analyzed with FlowJo. Gates were drawn according to cells stained only with the secondary antibodies and gates were maintained identical for all lines of different genetic backgrounds in each analysis.

### Cell cycle analysis

Analysis of the cell cycle profile was performed using the Click-iT EdU Pacific Blue Flow Cytometry Assay Kit (Thermo Fisher Scientific) according to the manufacturer's instructions. Cultured cells were treated for 1 hr with 10 μM EdU at 37°C and then harvested using TrypLE (GIBCO). About $10^6$ cells of each line were carried forward for staining. After washing three times with PBS/1% BSA, cells were fixed with 4% paraformaldehyde at room temperature for 15 min and washed again thrice with PBS/1% BSA. Cells were permeabilized with saponin-based permeabilization/wash buffer for 15 min and incubated with the Click-iT reaction cocktail for 30 min. Cells were washed once with permeabilization/wash buffer and then stained for DNA content using the FxCycle Far Red dye (Invitrogen). Cells were analyzed on a BD FACSCantoII flow cytometer and FlowJo software.

### Quantitative PCR

Total RNA was extracted with the Sigma GeneElute Total RNA kit (RTN350). The on-column DNase digestion step was performed (DNASE70, Sigma) to remove any genomic DNA contamination. 500ng of total RNA was used to synthesize cDNA with SuperScript II (Life Technologies) using Random primers (Promega C1181) and following the manufacturers instructions. cDNA was diluted 30 fold and 2.5uL was used to perform Quantitative PCR using Kapa SYBR fast Low-Rox (Sigma KK4622) in a final reaction volume of 7.5uL on a QuantStudio 5 384 PCR machine (ThermoFisher). Samples were run in technical duplicate and results were analyzed using *Gapdh* as the housekeeping gene. All primer pairs were validated to ensure only one product and a PCR efficiency of 100% (+/− 10%). Primer sequences used are displayed in Table S1.

### Cellular Fractionation

Cells were washed once with PBS and dissociated with Cell Dissociation Buffer (GIBCO) for 5 mins at 4C. Cells were collected and washed once with ice cold D-PBS. Pellets were resuspended in 5 x packed cell volume (PCV) of Isotonic Lysis Buffer (ILB; 10mM Tris-HCl, 3mM CaCl, 2mM MgCl2, 0.32M Sucrose, pH 7.5) supplemented with protease and phosphatase inhibitors (Roche 05892970001 PHOS-RO) and incubated on ice for 10 mins. 0.1% Triton X-100 was added and cells were incubated for a further 3 mins before being centrifuged at 1.5 K rpm for 3 mins. The supernatant (cytoplasmic enriched fraction) was removed and stored for downstream analysis. The pellet was washed once with 10 x PCV of ice cold ILB and resuspended in 2 x PCV of Nuclear Lysis Buffer (NLB; 50mM Tris-HCl, 150mM NaCl, 1% Triton X-100, 0.5% sodium deoxycholate, 0.1% SDS, pH 8.0) supplemented with PPI. The nuclei were broken open by dounce homogenization on ice and incubated for 30mins at 4C on a rotating wheel. 125u/mL Benzonase Nuclease (Sigma E8263) was added and the nuclei incubated for a further 45 mins at room temperature. Nuclei were centrifuged at 108K G for 30 mins at 4C and the supernatant (soluble nuclear enriched fraction) was stored for further downstream analysis. Protein concentration was determined by BCA assay (Pierce 23227). Fractionation was assessed by western blot for appropriate cell compartment markers.

### Western Blot

For whole cell lysates, cells were washed once in D-PBS and then resuspended in ice cold RIPA buffer (150mM NaCl, 50mM Tris, pH8.0, 1% NP-40, 0.5% sodium deoxycholate, 0.1% sodium dodecyl sulfate) containing protease and phosphatase inhibitors for 10 mins. Protein amount was quantified by dilution 1:4 in D-PBS, 10uL was measured in technical duplicate by BCA assay (Pierce 23227) following the manufacturer's instructions using a standard curve generated from BSA and read at 600nm on an EnVision 2104 plate reader). Samples were prepared by adding 4x NuPAGE LDS sample buffer (Life Technologies NP0007) plus 1% B-mercaptoethanol and heated at 95C for 5 mins. 5-10ug of protein per sample was run on a 4%–12% NuPAGE Bis-Tris Gel (Life Technologies) and then transferred to PVDF membrane by liquid transfer using NuPAGE Transfer buffer (Life Technologies, NP0006). Membranes were blocked for 1hr at room temperature in PBS 0.05% Tween-20 (PBST) supplemented with 4% non-fat dried milk and incubated overnight at 4C with primary antibodies diluted in the same blocking buffer. After three washes in PBST, membranes were incubated for 1h at RT with horseradish perosidase (HRP)-conjugated secondary antibodies diluted in blocking buffer, then washed a further three times before being incubated with Pierce ECL2 Western Blotting Substrate (ThermoFisher) and expsed to X-Ray Film. Membranes were probed with antibodies against active β-catenin (1:2000, Millipore 05-665, RRID: AB_309887), total β-catenin (1:2000, R&D AF1329, RRID: AB_354736), Otx2 (1:2000, R&D AF1979, RRID: AB_2157172), Nanog (1:500, R&D AF1997, RRID: AB_355097), Histone H3 (1:10000, Sigma H0164, RRID: AB_532248) and alpha-tubulin (1:10000, Sigma T6199, RRID: AB_477583).

### Immunocytochemistry

Cells were washed once with D-PBS before being fixed for 20 mins with 4% PFA. Cells were permeabilised and blocked in PBS 0.1% Triton X-100 (Sigma) and 5% donkey serum (Bio-Rad) for 30 minutes. Antibodies (primary and secondary) were incubated in PBS 0.1% Triton X-100 (Sigma) and 1% donkey serum for staining. Primary antibody was incubated for 3 hours at room temperature and cells were washed three times before incubating with secondary antibody for 2 hours. Cells were washed three times, incubated with DAPI for 20 minutes and kept in PBS 1%BSA for imaging. Images were colored and merged where indicated in the figure legend using ImageJ software (RRID: SCR_003070).

### Luciferase Reporter Assay

Cells were split in single cells with TrypLE 20 000 cells were plated per well of a 96-well plate. 500 ng of TOP Flash luciferase (Addgene vector 12456, RRID: Addgene_12456) and 50 ng of Renilla luciferase (Promega) were transfected with 3 μl of GeneJuice in 100μl of OptiMEM basal media supplemented with 10ng/ml LIF, 3 μM CHIR99021 and 1 μM PD0325901. Cells were grown over night and then either put in 2iLIF media or N2B27 basal media for 24 hours.

Cells were washed once with D-PBS, lysed in 90uL of 1 x Passive Lysis Buffer (Promega E1910) and incubated, shaking, for 10mins at room temperature. Insoluble debris was pelleted by centrifugation at 10K rpm for 5min at 4C and 20uL of the supernatant was transferred to a white, flat bottomed 96 well plate (Greiner, 655075) for analysis of Firefly and Renilla luciferase activity using the dual luciferase reporter assay system (Promega E1910) on a Glomax luminometer with dual injectors following the manufacturer's instructions.

### ELISA Assay

2.5ug of total protein from each cellular enriched fraction (described above) was diluted in 100uL of standard dilution buffer and assayed for total β-Catenin amount by ELISA following the manufacturer's instructions (Life Technologies KHO1211). Each sample was run in technical duplicate and quantified against a standard curve of total β-Catenin. The assay was read at 450nm on an EnVision 2104 reader (Perkin Elmer).

### Bulk RNA-seq on inbred strain mESCs

RNA-seq was performed on inbred mESC lines as described in Skelly et al. Briefly, mESCs were harvested at P6-P8 after removing MEFs by sequential plating; total RNA was extracted using the RNeasy (QIAGEN) RNA extraction kit; poly(A) RNA-seq libraries were constructed using the TruSeq Stranded mRNA Library Prep Kit (Illumina); barcoded libraries were then pooled and sequenced 125 bp paired-end on the HiSeq2500 (Illumina) using TruSeq SBS Kit v4 reagents. Sample processing and RNA sequencing were performed in two batches; C57BL/6J and PWD/PhJ cell lines were processed in the first batch followed by 129S1/SvImJ and CAST/EiJ backgrounds in a later batch. Analysis of the resulting raw fastq files followed Skelly et al. Briefly, sample fastq files were aligned against strain-specific transcriptomes using the bowtie aligner (Langmead et al., 2009), and then isoform-level and gene-level expression abundances were estimated with EMASE (Raghupathy et al., 2018). Read counts were estimated in this manner on both raw and batch corrected data. Further analyses were conducted on both raw and batch corrected read count data to identify and mitigate batch effects. To correct for batch effects, the first principal component was regressed out from the raw transcript counts, and the data was log transformed prior to principal component analysis. Finally, DESeq2 (Love et al., 2014) was used to test for variation in gene expression driven by genetic background.

### Expression QTL Mapping

Expression quantitative trait loci (eQTL) were mapped using RNA-seq data from 183 fully genotyped Diversity Outbred mESC lines as detailed in Skelly et al. Briefly, mESC lines were derived at Predictive Biology, Inc. (Carlsbad, CA) and maintained in 1i+LIF medium; genomic DNA for each line was extracted and genotyped at 144k SNV markers on the Giga Mouse Universal Genotyping Array platform (GeneSeek Neogen); total RNA was extracted and stranded libraries were prepared by AKESOgen using the TruSeq Stranded mRNA HT kit (Illumina); barcoded libraries were pooled and sequenced on the NextSeq platform, yielding 6M-55M 2x75bp paired-end reads per sample; only the first read of the pair was used in the downstream quantitation and eQTL mapping analyses. For expression analysis, single-end 75bp reads were aligned with BWA (Li and Durbin, 2009) to a pooled "8-way" transcriptome containing strain-specific isoform sequences from all eight inbred founder strains; EMASE (Raghupathy et al., 2018) was used to resolve multi-mapping reads and estimate transcript- and gene-level abundance estimates; gene-level raw counts were normalized to the upper quartile value in each sample to account for differences in library size, followed by batch correction using ComBat (Leek et al., 2012) and transformation to rank normal scores using r/DOQTL (Gatti et al., 2014). Finally, eQTL mapping was performed on normalized, transformed gene-level expression values using the 'scan1' function in r/qtl2 (Broman et al., 2006) with sex included as an additive covariate in the mapping model. This regression model estimates the additive effects of each of the eight DO founder strain haplotypes at each tested SNP on the expression levels of the gene of interest. For the founder haplotype analysis of the Axin2 eQTLs in Figure 4G, these founder strain effects for B6, CAST, and PWK at the Chr 3 and Ch 5 peaks estimated from the DO mESCs were compared to Axin2 expression measured by RNA-seq in the founder mESC lines (note - PWK founder coefficient compared to PWD RNA-seq).

### Single Cell analysis

Single-cell RNA-seq libraries were prepared in the Cancer Research UK Cambridge Institute Genomics Core Facility using the following: Chromium Single Cell 3′ Library & Gel Bead Kit v3, Chromium Chip B Kit and Chromium Single Cell 3′ Reagent Kits v3 User Guide (Manual Part CG000183 Rev A; 10X Genomics). Suspensions were loaded on the Chromium instrument with the expectation of collecting up to 4500 gel-beads emulsions containing single cells. RNA from the barcoded cells for each sample was subsequently reverse-transcribed in a C1000 Touch Thermal cycler (Bio-Rad) and all subsequent steps to generate single-cell libraries were performed according to the manufacturer's protocol with no modifications (12 cycles used for cDNA amplification). cDNA quality and quantity was measured with Agilent TapeStation 4200 (High Sensitivity 5000 ScreenTape) after which 25% of material was used for gene expression library preparation.

Library quality was confirmed with Agilent TapeStation 4200 (High Sensitivity D1000 ScreenTape to evaluate library sizes) and BMG LABTECH Clariostar Monochromator Microplate Reader (Invitrogen Quant-iT dsDNA Assay Kit; high sensitivity to evaluate dsDNA quantity). Each sample was normalized and pooled in equal molar concentration.

Pool was sequenced using 1 lane of SP flowcell on Illumina NovaSeq6000 sequencer with following parameters: 28 bp, read 1; 8 bp, i7 index; and 91 bp, read 2.

### Demultiplexing of 10x Data

Genotyping information for the C3H_HeJ, CAST_EiJ and C57BL_6NJ mouse strains were extracted from the Mouse Genome Project (Keane et al., 2011) dataset. The SNPs were filtered to identify those which w ere heterozygous in at least one of the three strains (25.7 million in total). These were used as candidates to genotype all of the cells in each pool using cellSNP v0.1.7 (Huang et al., 2019), parameters "–minMAF 0.1–minCOUNT 20." 76,000 to 111,000 informative SNPs were obtained from the pooled scRNA-seq data, these were utilized further in Vireo v0.2.2 (Huang et al., 2019) in the genotype reference free mode with parameters "-N 4 -M 100" to de multiplex the pools. The estimated genotypes for these strains were mapped back to the three known genotypes from the Mouse Genome Project to link the cell lines to their parental mouse strain.

### Analysis of 10x Data

Fastq files were aligned with the software Cell Ranger, using the pre-built mouse (mm10) reference available from 10X. The dataset was augmented with mouse embryonic fibroblasts (MEFs) as a negative control (Zhao et al., 2018). Read counts were imported into the Scanpy package (Wolf et al., 2018, RRID: SCR_018139). Genes with read counts > 0 in at least 3 cells were kept for downstream analysis. Low quality cells were filtered out based on the percentage of UMI mapping to the mitochondrial genome and the doublet annotation identified by Vireo (Huang et al., 2019). Normalization and identification of highly variable genes were performed using the Scanpy package. The normalized data were clustered using the Louvain method. Transcriptional similarity was quantified by estimating the connectivity of data manifold partitions within the partition-based graph abstraction (PAGA) framework (Wolf et al., 2018). Strain markers and differentially expressed genes between collection time points were identified by applying the Wilcoxon-Rank-Sum test ($p$ value < 0.01, |log2 fold change| > 1). Gene ontology was performed using TopGO (Alexa and Rahnenführer, 2019, RRID: SCR_014798).

## QUANTIFICATION AND STATISTICAL ANALYSIS

Unpaired Students Two Tailed t test was used (assuming equal variance) in excel for statistical analysis of all flow cytometry, quantitative PCR, luciferase assay and ELISA data. Details of the statistical analysis including exact value of n, what n represents, error bar description and reported p values can be found in the corresponding figure legends.

Cell Stem Cell, Volume *27*

## Supplemental Information

# Naive Pluripotent Stem Cells Exhibit Phenotypic Variability that Is Driven by Genetic Variation

**Daniel Ortmann, Stephanie Brown, Anne Czechanski, Selcan Aydin, Daniele Muraro, Yuanhua Huang, Rute A. Tomaz, Anna Osnato, Giovanni Canu, Brandon T. Wesley, Daniel A. Skelly, Oliver Stegle, Ted Choi, Gary A. Churchill, Christopher L. Baker, Peter J. Rugg-Gunn, Steven C. Munger, Laura G. Reinholdt, and Ludovic Vallier**

Supplementary information:

Figure S1.
Genetic background defines the transcriptional profile of naïve mESC. Related to Figure 1.

A

| Strain | Cell Line | Karyotype | Germline Transmission |
|---|---|---|---|
| PWD/PhJ | 401 | 70% XY | 63% |
| PWD/PhJ | 403 | 70% XY | 22% |
| PWD/PhJ | 398 | 70% XY | n/a |
| CAST/EiJ | 77 | 74% XY | 100% |
| CAST/EiJ | 78 | 81% XY | 100% |
| CAST/EiJ | 80 | 80% XY | 100% |
| C57BL/6J | 49 | 95% XY | 100% |
| C57BL/6J | 139 | 100% XY | n/a |
| C57BL/6J | 146 | 95% XY | n/a |
| C3H/HeJ | 386 | 80% XY | 90% |
| C3H/HeJ | 388 | 77% XY | 81% |
| C3H/HeJ | 389 | 67% XY | n/a |

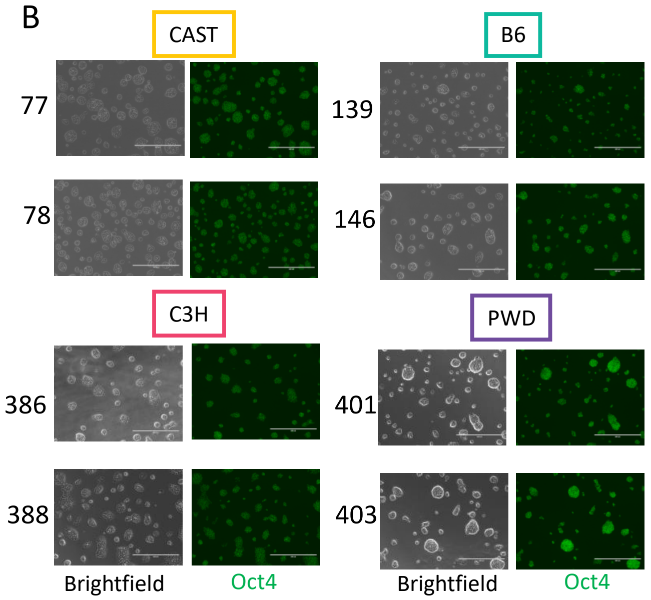


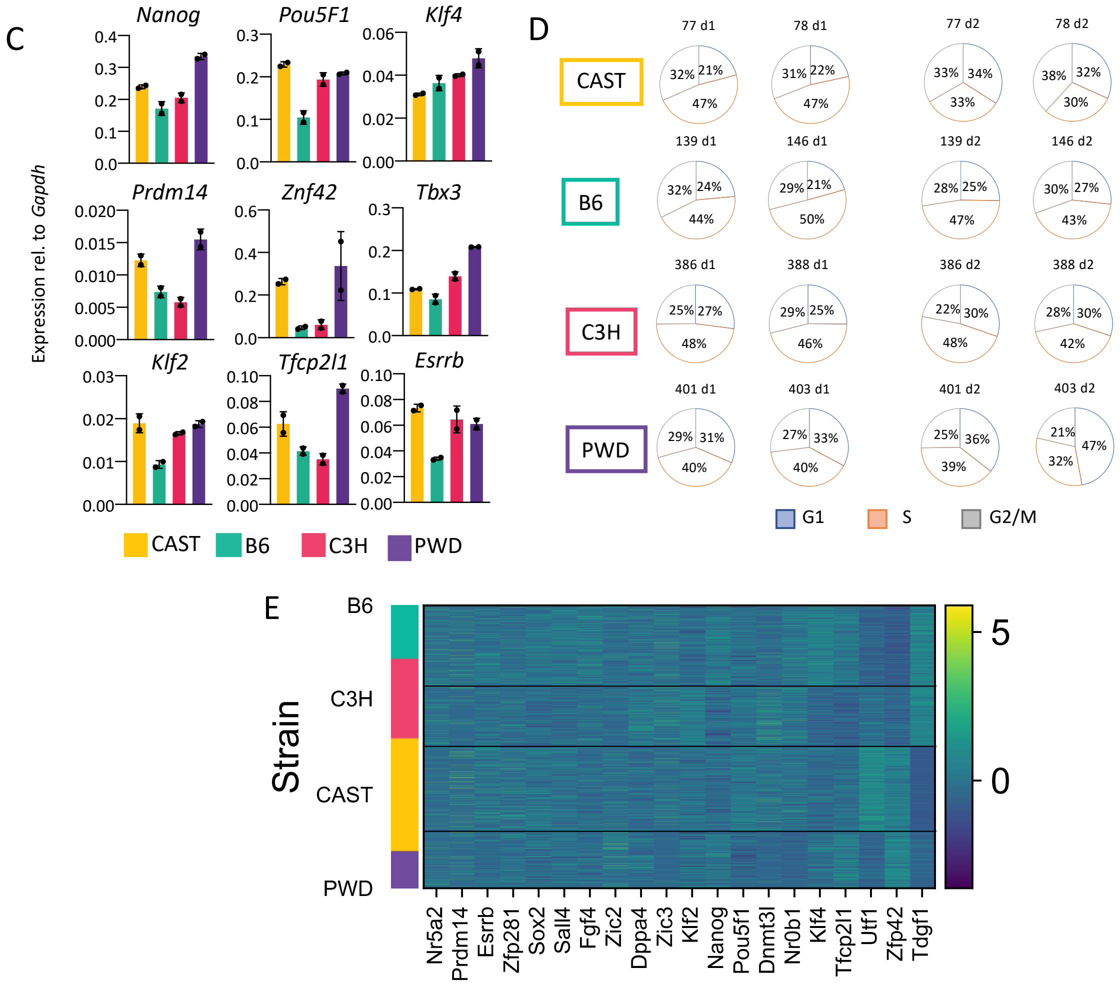

Supplementary figure 1 legend

A. Karyotype and chimera formation information for the lines used in this study.
B. Representative bright field and immunocytochemistry images of cells growing in 2iL. Number indicates the specific line shown.
C. Gene expression analysis for selected ground state/pluripotent markers. Data shown are the mean of 2 replicates per genetic background, 1 line per background. Error bars represent standard deviation of the means.
D. Cell cycle profiles over 2 days in 2iL condition. Data shown are representative of two independent experiments. Number indicates the specific line shown.
E. Heat map of selected z-scored normalised markers generated from Single Cell transcriptomic data.

Figure S2.

Genetic background influences early differentiation capacity of naïve mESCs. Related to Figure 2.

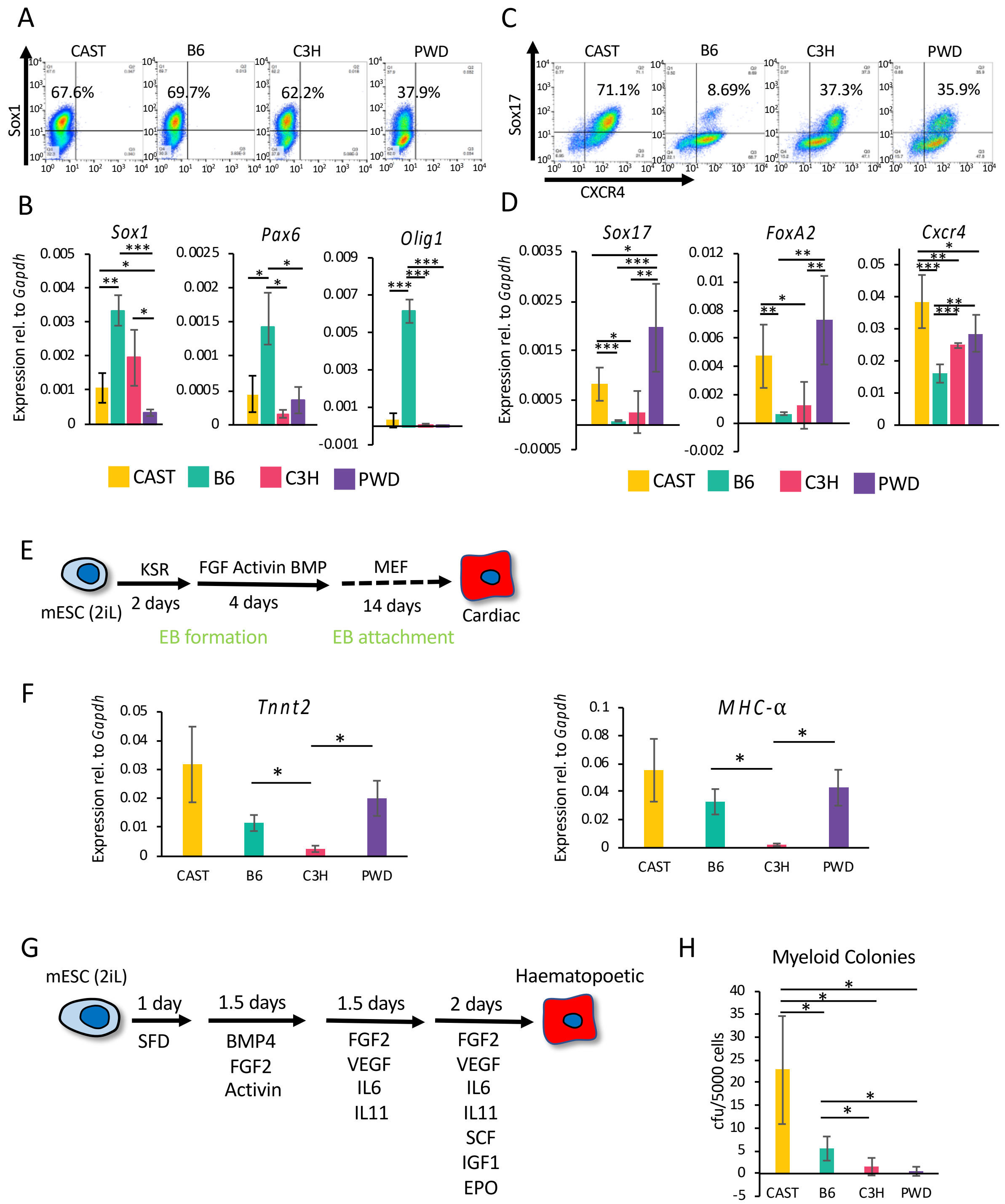

Supplementary Figure 2 Legend

A. Representative flow cytometry analysis of SOX1 after 6 days of undirected differentiation.
B. Gene expression analysis of key neural markers after 2 days of undirected differentiation. Data shown are from 2 differentiations for each genetic background, 2 lines per background. Error bars represent standard deviation of the means. Two tailed T-test *$p<0.05$, **$p<0.005$, ***$p<0.0005$.
C. Representative flow cytometry analysis of SOX17 and CXCR4 after 6 days of differentiation towards endoderm.
D. Gene expression analysis of key endodermal markers after 6 days of directed differentiation towards endoderm. Data shown are from 2 independent differentiations for each genetic background, 2 lines per genetic background. Error bars represent standard deviation of the means. Two tailed T-test *$p<0.05$, **$p<0.005$, ***$p<0.0005$.
E. Schematic representation of the cardiac differentiation process.
F. Gene expression analysis of key cardiac markers at the end of the differentiation process. Data shown are from 2 independent differentiations for each genetic background, 2 lines per genetic background. Error bars represent standard errors of the means. Two tailed T-test *$p<0.05$.
G. Schematic representation of the haematopoetic differentiation process.
H. Colony forming assay assessing haematopoetic differentiation. Data shown are from 1 differentiation for each genetic background, 2 lines per genetic background. Error bars represent standard deviation of the means. Two tailed T-test *$p<0.05$.

Figure S3.

Transition from mESCs to EpiLCs varies between genetic backgrounds. Related to Figure 3.

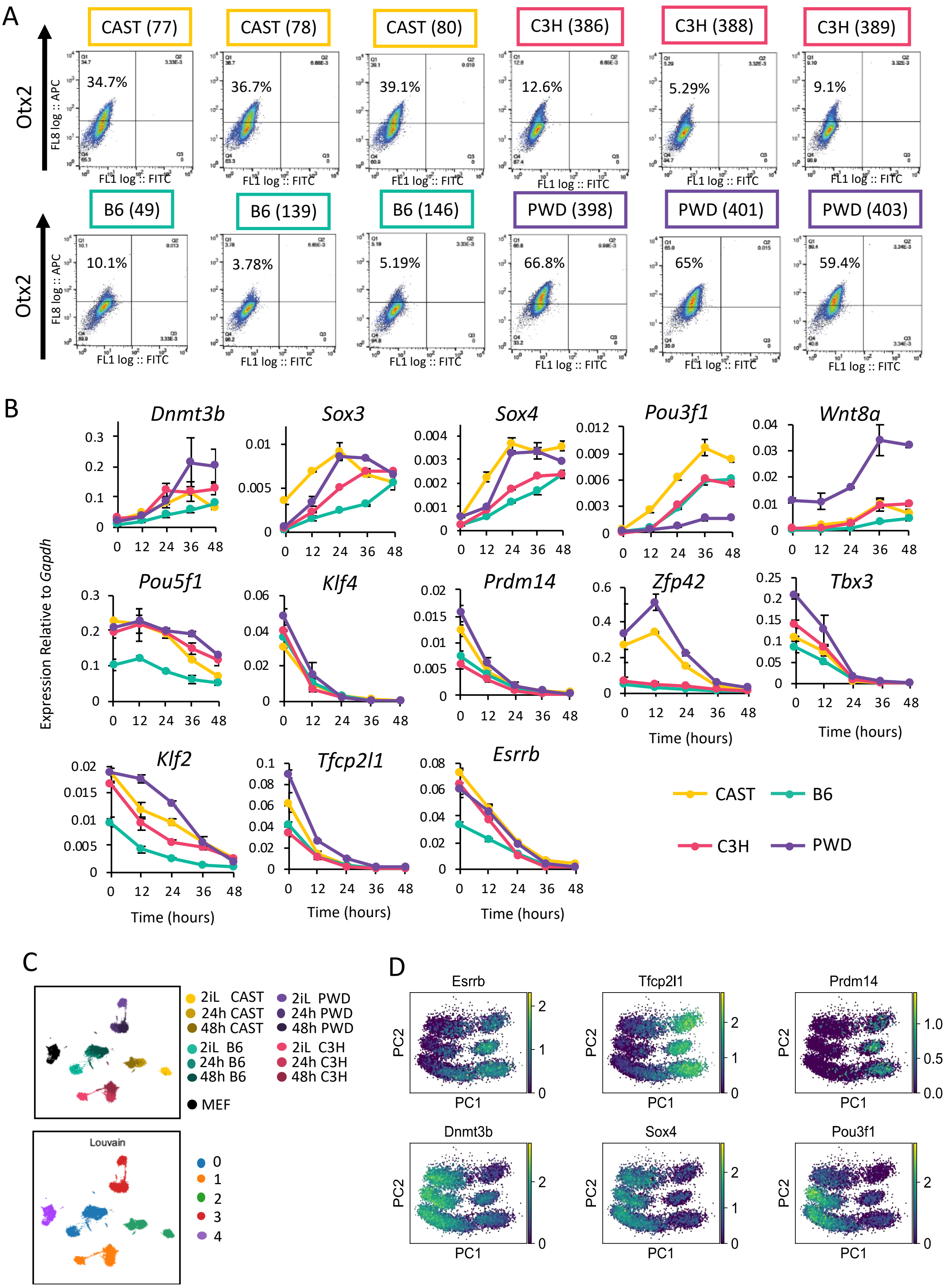

Supplementary figure 3 legend

A. Flow cytometry plots for OTX2 after 2 days of undirected differentiation in N2B27. Plots shown are from 1 differentiation, number in brackets indicates the specific line shown.
B. Gene expression analysis of selected markers during 2 days of undirected differentiation in N2B27. Error bars represent standard deviation of the means, 1 line per genetic background.
C. UMAP and Louvain clustering using 300 PCs (51.2% variance explained) during 2 days of undirected differentiation in N2B27. 5 clusters partition strains and negative control (MEF).
D. Log-normalised gene expression values of selected differentiation markers.

Figure S4.
Signalling activity is influenced by genetic background. Related to Figure 4.

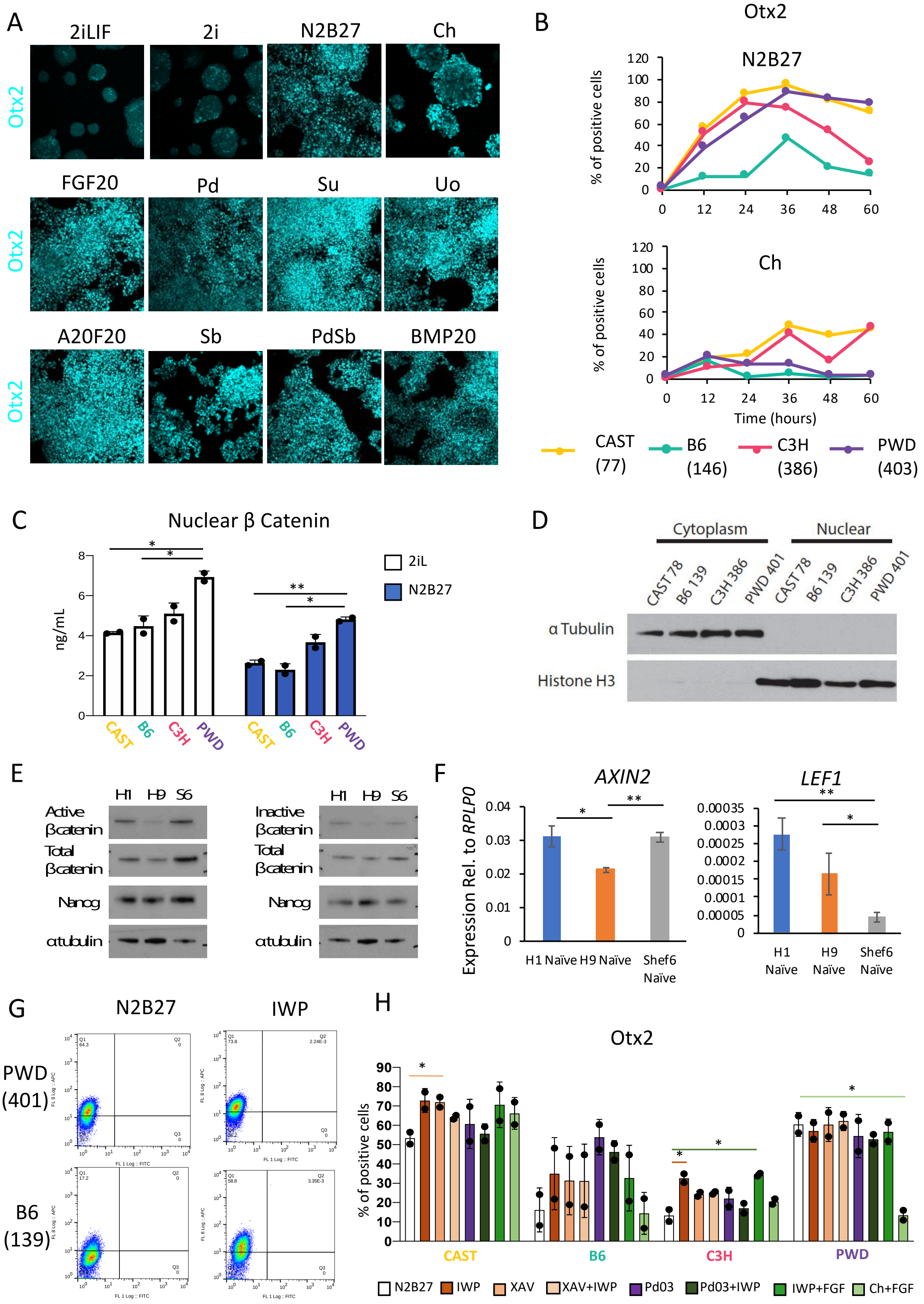

Supplementary Figure 4 Legend

A. Representative immunocytochemistry images for Otx2 after 2 days in the indicated culture conditions. Images shown are from the CAST background.
B. Quantification of flow cytometry analysis for Otx2 over a 60 hour time course in the indicated culture conditions. Number in brackets indicates the line shown.
C. ELISA for total β Catenin in a nuclear enriched cellular fraction after 1 day in the indicated culture conditions. Data shown are the mean from 2 lines per genetic background. Error bars represent standard deviation of the means. Two tailed T-test *$p<0.05$, **$p<0.005$.
D. Western blot for cytoplasmic and nuclear markers after cell fractionation. Number in brackets indicates the line shown.
E. Western blot for active and total β Catenin in human naive state cells derived from 3 different genetic backgrounds.
F. Gene expression analysis of selected WNT signalling target genes in human naïve state cells. Data shown are the mean from 3 replicates for each genetic background. Error bars represent standard deviation of the means. Two tailed T-test *$p<0.05$, **$p<0.005$.
G. Representative flow cytometry plots for Otx2 after 2 days in the indicated culture conditions. Number in brackets indicates the line shown.
H. Quantification of flow cytometry analysis for Otx2 after 2 days in the indicated culture conditions. Data shown are the mean from 2 lines per genetic background. Error bars represent standard deviation of the means. Two tailed T-test *$p<0.05$.

Table S1.

a. Quantitative PCR primer sequences. Related to STAR Methods section.
b. PAGA analysis of single cell RNA-seq time course. Related to Figure S3c.

Supplementary table 1a:

| **Gene Symbol** | **Forward Primer (5'-3')** | **Reverse Primer (5'-3')** |
|---|---|---|
| *Gapdh* | ACACATTGGGGGTAGGAACA | AACTTTGGCATTGTGGAAGG |
| *Zfp42* | CAAGGAGGAAATAGGTAGAGCGCA | ACTTGGAGGCAGCACAGTGA |
| *Nanog* | CACAGTTTGCCTAGTTCTGAGG | GCAAGAATAGTTCTCGGGATGAA |
| *Pou5F1* | ATCACTCAATCCGCCAATCAGC | GCCGGTTACAGAACCATACTCG |
| *Sox2* | CACATGGCCCAGCACTACC | CACATGTGCGACAGGGGC |
| *Klf4* | AAGAACAGCCACCCACACTT | GGTAAGGTTTCTCGCCTGTG |
| *Prdm14* | GCATATACCCTACCCGCTTTC | CAAACGGATTGGAGGTTGAT |
| *Otx2* | TATCTAAAGCAACCGCCTTACG | AAGTCCATACCCGAAGTGGTC |
| *Dnmt3b* | GCCCATGCAATGATCTCTCT | CCAGAAGAATGGACGGTTGT |
| *Pou3F1* | GATCCAGAATGCGCCAACTCAC | CCTCTCTTCGTCCATTCTCCCG |
| *FGF5* | GGGATTGTAGGAATACGAGGAGTT | TGGCACTTGCATGGAGTTT |
| *Sox1* | TGTAATCCGGGTGTTCCTTC | AGTGGAAGGTCATGTCCGAG |
| *Axin2* | GAGAGTGAGCGGCAGAGC | CGGCTGACTCGTTCTCCT |
| *Lef1* | TCCTGAAATCCCCACCTTC | ACCCGTGATGGGATAAACAG |
| *Wnt5a* | CTGGAGGTGCCATGTCTTCC | TCGGCTGCCTATTTGCATCA |
| *Pdgrfa* | AAGACCTGGGCAAGAGGAAC | GAACCTGTCTCGATGGCACT |
| *Gata6* | GGTCTCTACAGCAAGATGAATGG | TGGCACAGGACAGTCCAAG |
| *Sox7* | GCGGAGCTCAGCAAGATG | GGGTCTCTTCTGGGACAGTG |
| *Lama1* | AGGTCTGCGTTGAGTGTTCTG | CAGTACTATGCCGTCAGCGAT |
| *Sparc1* | AGGGCCTGGATCTTCTTTCTC | CAAATTCTCCCATTTCCACCT |
| *Tbx3* | CTGCCCTTCCACCTCCAACA | GGGGCGCATGCTGTTCAAAT |
| *Klf2* | AAGAGCTCGCACCTAAAGGC | CGCATCCTTCCCAGTTGC |
| *Tfcp2l1* | GGGGACTACTCGGAGCATCT | TTCCGATCAGCTCCCTTG |
| *Esrrb* | GATGCTGAAGGAAGGTGTGC | GGCTTTTTAGCAGGTGGGGA |
| *Tnnt2* | CAACATGATGCACTTTGGAGGGT | TCGCAGAACGTTGATTTCGTATT |
| *MHC alpha* | GCCCAGTACCTCCGAAAGTC | GCCTTAACATACTCCTCCTTGTC |
| *FoxA2* | CCCTACGCCAACATGAACTCG | GTTCTGCCGGTAGAAAGGGA |
| *Cxcr4* | CTTCTGGGCAGTTGATGCCAT | CTGTTGGTGGCGTGGACAAT |
| *Sox3* | CTGGAAACTGCTGACCGATG | TCCGGGTACTCCTTCATGTG |
| *Sox4* | CTCAAGGACAGCGACAAGATTCC | AGCCATGTGCTTGAGGCG |
| *Wnt8a* | CATGTACGCAGTCACCAAGAA | CATCCTTCCCTTTCTCCAAAC |
| hs *AXIN2* | GCTCTGTTTGTCTTAAAGGTCT | AGGAACTGTCATTTCCACGAAAG |
| hs *LEF1* | TGAGCCTCGAGAAGAAAAACCG | CCACGCTGGAGATGTCCGTT |
| hs *RPLP0* | CAACTGTTGCATCAGTACCCCATTCT | ACTCTTCCTTGGCTTCAACCTTAGCT |
| | | |
| *Sox17* | QuantiTect Mm_Sox17_1_SG | QT00160720 |

Supplementary Table 1b:

| | 2iLCAST | 24hCAST | 48hCAST | 2iLB6 | 24hB6 | 48hB6 | 2iLPWD | 24hPWD | 48hPWD | 2iLC3H | 24hC3H | 48hC3H | MEF |
|---|---|---|---|---|---|---|---|---|---|---|---|---|---|
| 2iLCAST | 0 | 4.43 | 1.09 | 0.37 | 0 | 0 | 2.92 | 0.19 | 0 | 0 | 0 | 0.18 | 0.56 |
| 24hCAST | 4.43 | 0 | 100 | 0.14 | 0.3 | 0.67 | 0.48 | 1.88 | 0.28 | 0 | 0 | 0.39 | 0 |
| 48hCAST | 1.09 | 100 | 0 | 0 | 0 | 0.72 | 0.7 | 1.558 | 0.22 | 0 | 0 | 2.28 | 0 |
| 2iLB6 | 0.37 | 0.14 | 0 | 0 | 13.66 | 6.94 | 0.32 | 0 | 0.23 | 0.67 | 0 | 0.32 | 0 |
| 24hB6 | 0 | 0.3 | 0 | 13.66 | 0 | 100 | 0 | 0.06 | 0.26 | 0.13 | 0.82 | 0 | 0 |
| 48hB6 | 0 | 0.67 | 0.72 | 6.94 | 100 | 0 | 1.05 | 3.04 | 0.08 | 0 | 1.46 | 8.11 | 0 |
| 2iLPWD | 2.92 | 0.48 | 0.7 | 0.32 | 0 | 1.05 | 0 | 14.97 | 9.33 | 0 | 0 | 0.46 | 0 |
| 24hPWD | 0/19 | 1.88 | 1.58 | 0 | 0.06 | 3.04 | 14.97 | 0 | 100 | 0.09 | 0.27 | 2.65 | 0 |
| 48hPWD | 0 | 0.28 | 0.22 | 0.23 | 0.26 | 0.08 | 9.33 | 100 | 0 | 0 | 0 | 0.26 | 0 |
| 2iLC3H | 0 | 0 | 0 | 0.67 | 0.13 | 0 | 0 | 0.09 | 0 | 0 | 6.48 | 2.67 | 0 |
| 24hC3H | 0 | 0 | 0 | 0 | 0.82 | 1.46 | 0 | 0.27 | 0 | 6.48 | 0 | 100 | 0 |
| 48hC3H | 0.18 | 0.39 | 2.28 | 0.32 | 0 | 8.11 | 0.46 | 2.65 | 0.26 | 2.67 | 100 | 0 | 0 |
| MEF | 0.56 | 0 | 0 | 0 | 0 | 0 | 0 | 0 | 0 | 0 | 0 | 0 | 0 |
